# Association of trilaciclib use with hematologic outcomes during perioperative immunochemotherapy in resectable esophageal squamous cell carcinoma: a prospective observational cohort study

**DOI:** 10.3389/fphar.2026.1933364

**Published:** 2026-10-28

**Authors:** Chaoquan He, Xirui Lin, Haijie Xu, Jiong Lin, Peishen Li, Weisen Wang, Bo Chen, Ruijun Lin, Shaobo Zhang, Haitao Li, Xiaoyu Zhang, Yu Li, Zexin Huang, Ruen Xie, Zefeng Chen, Jiaying Wu, Chenshu Huang, Rui Guo, Hongling Gao, Zefeng Xie, Hansheng Wu

**Affiliations:** 1 Department of Thoracic Surgery, The First Affiliated Hospital of Shantou University Medical College, Shantou, China; 2 Shantou University Medical College, Shantou, China; 3 Department of Thoracic Surgery, Cancer Hospital of Shantou University Medical College, Shantou, China

**Keywords:** esophageal squamous cell carcinoma, immunochemotherapy, myelosuppression, perioperative therapy, prospective observational cohort study, trilaciclib

## Abstract

**Background:**

Perioperative immunochemotherapy is a key treatment strategy for resectable esophageal squamous cell carcinoma (ESCC), but chemotherapy-induced myelosuppression (CIM) may compromise treatment delivery. Trilaciclib, a transiently acting, reversible cyclin-dependent kinase 4/6 (CDK4/6) inhibitor, has shown myeloprotective effects in advanced solid tumors; however, evidence in perioperative ESCC is limited. This study investigated the association between trilaciclib use and hematologic outcomes during perioperative immunochemotherapy.

**Methods:**

In this single-center, prospective observational cohort study, 93 patients (control group, n = 65; trilaciclib group, n = 28) enrolled between October 2023 and March 2026 were analyzed. Stabilized inverse probability of treatment weighting (IPTW) was used to balance baseline characteristics. Binary, longitudinal, and survival outcomes were analyzed using IPTW-weighted Firth-penalized logistic regression, weighted generalized estimating equations, and weighted Cox models, respectively, with false discovery rate (FDR) adjustment for multiple comparisons.

**Results:**

After weighting, baseline characteristics were well balanced (all standardized mean differences <0.10). Compared with control, the trilaciclib group had lower weighted incidences of grade 3–4 CIM (3.0% vs. 31.2%; odds ratio [OR] 0.11, 95% confidence interval [CI]: 0.01–0.48; FDR-adjusted *P* = 0.007), grade 3–4 neutropenia (3.0% vs. 28.3%; OR 0.12, 95% CI: 0.01–0.55; FDR-adjusted *P* = 0.010), and grade 3–4 anemia (0.0% vs. 17.3%; OR 0.08, 95% CI: <0.01–0.67; FDR-adjusted *P* = 0.030). Grade 3–4 thrombocytopenia, severe neutropenia, and febrile neutropenia did not differ significantly. Granulocyte colony-stimulating factor (G-CSF) and erythropoiesis-stimulating agent (ESA) use was also lower in the trilaciclib group (34.4% vs. 79.7% and 3.7% vs. 29.6%; FDR-adjusted *P* < 0.001 and *P* = 0.010, respectively). Overall survival, disease-free survival, and pathologic outcomes did not differ significantly. These findings should not be interpreted as evidence of equivalence or noninferiority.

**Conclusion:**

In this small, single-center cohort, trilaciclib use was associated with lower grade 3–4 myelosuppression and less hematopoietic supportive care. No statistically significant unfavorable differences in pathologic or survival outcomes were observed; however, equivalence cannot be inferred. These results are hypothesis-generating and require validation in randomized trials.

## 1 Introduction

Esophageal cancer (EC) is an aggressive gastrointestinal malignancy with high mortality and multifactorial pathogenesis (Jiang et al., 2025). According to GLOBOCAN 2022 and data from the National Cancer Center of China, China bears approximately half of the global burden of esophageal cancer in both incidence and mortality (Bray et al., 2024; Han et al., 2024). esophageal squamous cell carcinoma (ESCC) represents more than 90% of all esophageal cancer cases in China. At diagnosis, 60%–70% of patients present with locally advanced disease. Even after curative-intent surgery, the 5-year overall survival rate remains only 10%–40% (Yang et al., 2018).

In recent years, with the introduction of immune checkpoint inhibitors (ICIs), platinum-based chemotherapy combined with PD-1/PD-L1 inhibitors has gradually become one of the standard treatment modalities for locally advanced resectable ESCC (Yang et al., 2024; Yu et al., 2026). Perioperative immunochemotherapy may promote tumor downstaging, facilitate R0 resection, and eradicate micrometastatic disease, thereby increasing pathologic complete response (pCR) rates and potentially improving long-term outcomes (Zhang et al., 2025). The randomized phase 3 ESCORT-NEO trial and several comparative clinical studies have provided further evidence supporting the use of neoadjuvant immunochemotherapy (Qin et al., 2024; Guo et al., 2025; Wang et al., 2025). However, this approach relies heavily on cytotoxic chemotherapy, and myelosuppression represents one of its most frequent and clinically consequential hematologic toxicities. Qiao et al. reported that the incidence of myelosuppression during neoadjuvant immunochemotherapy (nICT) was as high as 68.8% in Chinese patients with ESCC (Qiao et al., 2022). Perioperative myelosuppression not only increases the risks of infection, bleeding, and febrile neutropenia (FN), but may also lead to treatment delays, dose reductions, or premature discontinuation, potentially compromising the completion of neoadjuvant therapy and timely progression to surgery (Crawford et al., 2024).

Current standard management of myelosuppression is mainly reactive, encompassing granulocyte colony-stimulating factor (G-CSF), erythropoiesis-stimulating agents (ESAs), and transfusion. These interventions are generally initiated after hematologic toxicity has already developed and are directed toward specific hematologic complications rather than providing broad multilineage myeloprotection (Crawford et al., 2024). In addition, immune checkpoint inhibitors themselves can cause hematologic immune-related adverse events, which are more difficult to distinguish and manage when they occur concurrently with chemotherapy-induced hematologic toxicity (Qiao et al., 2022). In the perioperative setting, where the goal is cure, proactive prevention of myelosuppression represents a potentially valuable complement to reactive supportive care and warrants prospective investigation.

Trilaciclib is a highly selective, reversible cyclin-dependent kinase (CDK) 4/6 inhibitor. By transiently inhibiting CDK4/6 activity at the G1/S checkpoint, trilaciclib prevents phosphorylation of the retinoblastoma (Rb) protein and induces temporary G1-phase arrest in HSPCs, thereby reducing cytotoxic injury to these cells during chemotherapy exposure and conferring myeloprotection (He et al., 2017). Prior studies in extensive-stage small-cell lung cancer (ES-SCLC), breast cancer, and other solid tumors have shown that trilaciclib reduces the risk of chemotherapy-induced myelosuppression (CIM) and decreases the need for hematopoietic supportive care (Ferrarotto et al., 2021). The Chinese phase 3 TRACES study and subsequent meta-analyses have reported broadly consistent findings (Cheng et al., 2024; Qiu et al., 2023). Its population pharmacokinetics and exposure–response relationship have also been well characterized (Li et al., 2023). Trilaciclib was first approved in 2021 and was subsequently approved by the National Medical Products Administration of China in July 2022 (Dhillon, 2021).

Despite this, evidence regarding trilaciclib use during perioperative immunochemotherapy for resectable ESCC remains limited, with only small real-world studies providing preliminary data on its feasibility (Chen et al., 2025; Yan et al., 2025). To address this evidence gap, in this prospective observational cohort study, we evaluated the association between trilaciclib use and hematologic outcomes among patients with resectable ESCC receiving perioperative immunochemotherapy. We also conducted exploratory analyses of its associations with pathologic response and survival outcomes, with the aim of generating hypotheses and informing the design of future randomized controlled trials.

## 2 Materials and methods

### 2.1 Study design and patients

This was a single-center, prospective, observational cohort study. We enrolled patients with histologically confirmed ESCC at the First Affiliated Hospital of Shantou University Medical College between October 2023 and March 2026 who received perioperative immunochemotherapy and were scheduled to undergo minimally invasive esophagectomy (MIE). The decision to administer trilaciclib was made by the treating physician based on the patient’s clinical condition, risk of hematologic toxicity, drug availability, and patient preference; treatment was not assigned by the investigators. Because treatment was not assigned according to a study protocol, the study was conducted as an observational rather than an interventional clinical trial. According to the International Committee of Medical Journal Editors (ICMJE) definition, this study is not a clinical trial subject to mandatory registration.

The inclusion criteria were as follows (1): age ≥18 years (2); histologically confirmedesophageal squamous cell carcinoma, clinical stage II-IVA (AJCCeighth edition) (3); scheduled to undergo MIE (4); planned to receive neoadjuvant and/or adjuvantplatinum-based chemotherapy, combined with PD-1/PDL1inhibitor therapy; and (5) willingness andability to participate in protocol-required assessments and followup.The exclusion criteria were as follows (1): historyof or concurrent other malignant tumors (2); distantmetastatic disease at diagnosis (3); receipt of any nonprotocol-specified anti-tumor therapy before enrollment (4); inability to complete key assessments; and (5) patients deemed unsuitable for inclusion byinvestigators.

Patient flow is shown in Figure 1. Of 120 patients assessed for eligibility, 97 were enrolled after 23 exclusions. As of the data cutoff date (30 March 2026), 78 patients had completed protocol-specified treatment and had evaluable overall survival (OS) and disease-free survival (DFS) data. Nineteen patients had incomplete follow-up: 15 had evaluable post-baseline hematologic data before hospital transfer (n = 10) or regimen change (n = 5) and were included in the safety analysis; the remaining four had no post-baseline data and were excluded. The final safety analysis set comprised 93 patients (65 in the control group and 28 in the trilaciclib group). Survival analysis included 78 patients; pathologic analysis included 52 nICT patients with evaluable surgical specimens. Timing, reasons, and distribution of incomplete follow-up are detailed in Supplementary Table S1.

**FIGURE 1:**
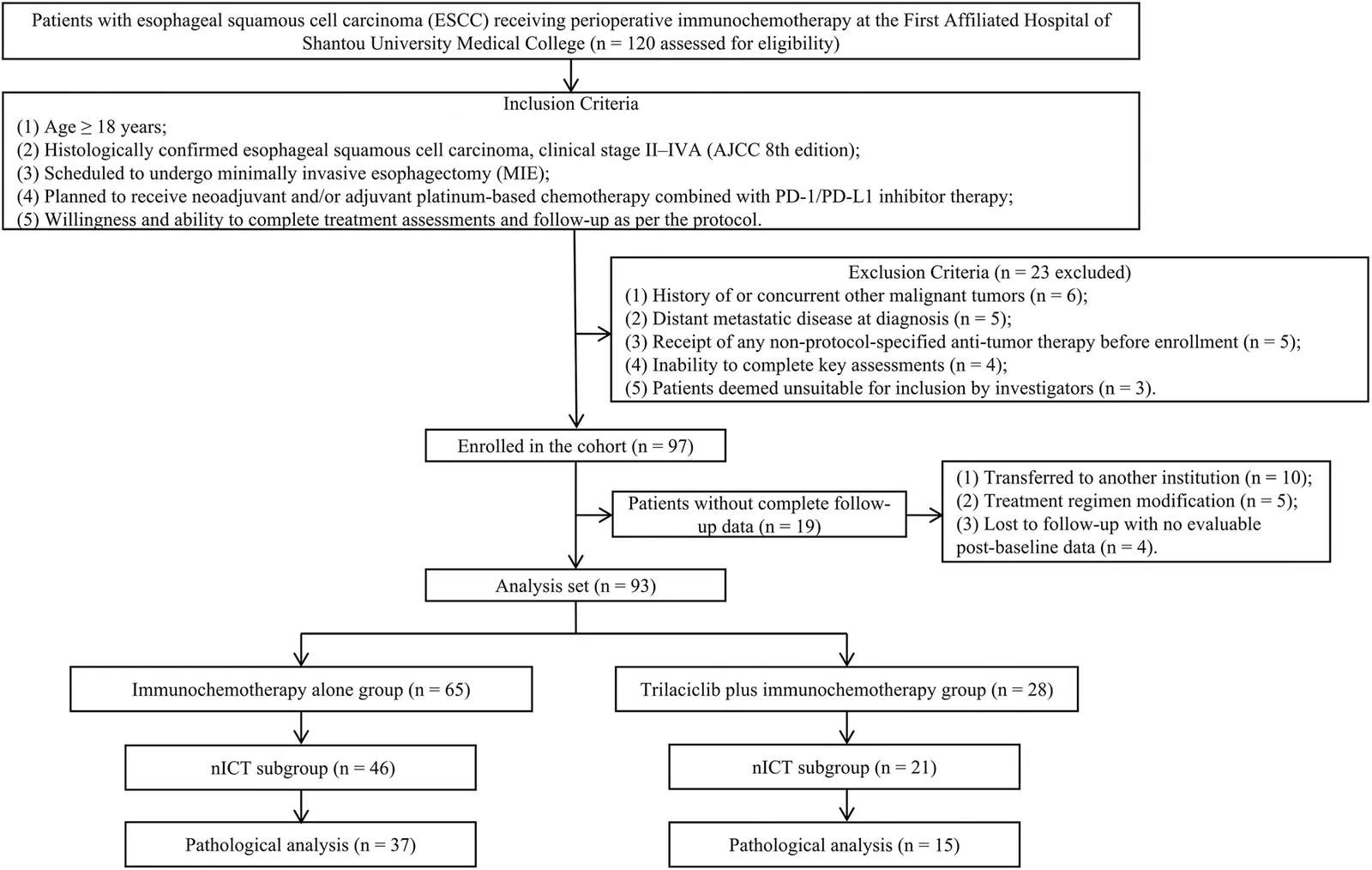
Flow of patient enrollment. Group assignment reflected routine clinical decisions by treating physicians; no intervention was assigned by the investigators. Safety/hematologic analyses used the 93-patient analysis set; survival analyses used the 78 patients with complete follow-up; pathological analyses used 52 nICT patients with evaluable surgical specimens. Further details are provided in Supplementary Table S1. Abbreviations: nICT, neoadjuvant immunochemotherapy.

Study data were prospectively collected via a prespecified case report form. All data were recorded in real time during treatment and follow-up. Data quality was verified against electronic medical records, laboratory findings, imaging studies, and pathology reports. Follow-up was conducted according to a predefined schedule, and the cutoff date for this analysis was 30 March 2026.

### 2.2 Treatment procedures

All patients received perioperative platinum-based doublet chemotherapy combined with a PD-1/PD-L1 inhibitor, administered in 3-week cycles. The most frequently used regimen was carboplatin plus albumin-bound paclitaxel (carboplatin AUC 5 mg·min/mL; albumin-bound paclitaxel 260 mg/m^2^). The specific PD-1/PD-L1 inhibitor and its dose were selected by the treating physician according to the patient’s clinical condition, prescribing information, and drug availability, with administration following the corresponding protocol.

In the trilaciclib group, trilaciclib (240 mg/m^2^) was administered via intravenous infusion within 4 h prior to chemotherapy in each cycle; the infusion lasted approximately 30 min. Subsequent chemotherapy and immunotherapy were delivered according to the predefined regimen. Patients in the control group did not receive trilaciclib; all other antitumor treatments and supportive care measures were delivered according to the same principles as those in the trilaciclib group.

During the neoadjuvant phase, patients received 2–4 cycles of immunochemotherapy prior to surgery. Upon completion of neoadjuvant therapy, resectability and surgical risk were assessed by imaging and endoscopy and subsequently reviewed by a multidisciplinary team (MDT). Patients deemed eligible for surgery underwent MIE with systematic lymph node dissection 4–6 weeks after the last treatment cycle. Adjuvant therapy was determined by the MDT based on pathological stage, margin status, lymph node metastasis, degree of pathological response, postoperative recovery, and patient preference.

Hematopoietic support included G-CSF, thrombopoietin (TPO), ESAs, and blood transfusion. Hematologic adverse events were graded according to the Common Terminology Criteria for Adverse Events, version 5.0 (CTCAE v5.0). Hematopoietic support was provided according to adverse event grade, clinical symptoms, laboratory findings, and clinical need. Identical supportive care principles were followed in both groups.

### 2.3 Outcome measures

The prespecified primary outcome was grade 3–4 CIM, analyzed as a binary outcome (grade 0–2 vs. grade 3–4) in the 93-patient safety analysis set. The primary analysis used IPTW-weighted Firth-penalized logistic regression. CIM was defined as a composite endpoint comprising leukopenia, neutropenia, anemia, and thrombocytopenia, with the CIM grade at each hematologic assessment defined as the highest grade observed among these four hematologic toxicities.

Hematologic assessments were performed 5–7 days after each immunochemotherapy administration and on the day before the subsequent cycle, and leukopenia, neutropenia, anemia, and thrombocytopenia were graded according to CTCAE version 5.0 (grading criteria are provided in Supplementary Table S2). Treatment cycles were numbered sequentially for each patient from the first immunochemotherapy administration (C1 onward); assessments obtained 5–7 days after administration were assigned to that cycle, and assessments obtained on the day before the subsequent cycle were assigned to the preceding cycle. The cycle-specific CIM grade was defined as the highest grade among the four toxicities observed during that cycle, and the patient-level CIM grade as the highest grade across all evaluable cycles; baseline blood counts were not included in the CIM assessment. Cycles without an available hematologic assessment were not assigned a cycle-specific grade. Patients with incomplete treatment or hematologic assessments were retained if evaluable post-baseline data were available: patients with at least one grade 3–4 CIM event during the evaluable period were classified as having an event, whereas those without a documented event were classified as having no observed event during that period (this classification applied only to the evaluable period and did not imply the absence of CIM thereafter); patients without evaluable post-baseline data were excluded.

Longitudinal analyses were exploratory. Because neoadjuvant and adjuvant cycles corresponded to pre- and postoperative periods that are not clinically comparable, the longitudinal exposure axis for the full cohort was reported descriptively only. The nICT subgroup, consisting entirely of preoperative cycles, served as a prespecified validation analysis.

Severe neutropenia (SN), defined as grade 4 neutropenia with an absolute neutrophil count (ANC) < 0.5 × 10^9^/L, and febrile neutropenia (FN), defined as an ANC <1.0 × 10^9^/L accompanied by fever, were also assessed. Secondary outcomes included G-CSF, TPO, and ESA use; transfusion support; treatment delay attributable to hematologic toxicity; nonhematologic adverse events; pathologic outcomes, including the R0 resection rate, pathologic complete response (pCR), major pathologic response (MPR), and postoperative pathologic stage; and disease-free survival (DFS) and overall survival (OS). Treatment delay attributable to hematologic toxicity was defined as postponement of the subsequent treatment cycle by more than 7 days beyond the protocol-specified planned date because of a hematologic adverse event, regardless of whether the delay occurred during the neoadjuvant or adjuvant phase. Nonhematologic adverse events were summarized by system organ class. OS and DFS were exploratory endpoints and were not subjected to multiplicity adjustment.

pCR was defined as the absence of residual viable tumor in both the primary tumor bed and the dissected lymph nodes. MPR was defined as ≤10% residual viable tumor in the primary tumor bed, including pCR. Pathologic assessments were independently performed by two pathologists blinded to treatment allocation. In cases of disagreement, a third senior pathologist participated in the discussion and reached a final consensus diagnosis. Lymph node status was incorporated into pathologic staging. DFS and OS were calculated from the date of surgery to recurrence, progression, or death and to death, respectively. Patients without an event were censored at the date of their last documented follow-up.

### 2.4 Statistical analysis

Continuous variables are presented as the median (Q1, Q3), and categorical variables are presented as numbers and percentages. Given the nonrandomized study design, stabilized inverse probability of treatment weighting (IPTW), targeting the average treatment effect in the overall population, was used to balance baseline characteristics and reduce confounding due to treatment selection.

Propensity scores were estimated using multivariable logistic regression. The model for the overall cohort included 12 prespecified covariates: age, Eastern Cooperative Oncology Group (ECOG) performance status, sex, body mass index, smoking status, alcohol use, tumor location, clinical TNM stage, and baseline leukocyte, neutrophil, hemoglobin, and platelet counts. The nICT subgroup was reweighted using a simplified five-covariate model that included age, performance status, TNM stage, tumor location, and baseline platelet count. All covariates were selected *a priori* based on clinical relevance. Multicollinearity was assessed before model fitting. The weights were truncated at the 1st and 99th percentiles.

Covariate balance was considered acceptable when the absolute standardized mean difference (SMD) was <0.10 and was displayed using Love plots (Figure 2; Supplementary Figure S3). Propensity score distributions and the region of common support are shown in Supplementary Figure S1. Weight distributions, extreme weights, and the effective sample size (ESS), calculated as ESS = (Σw)^2^/Σw^2^, are shown in Supplementary Figure S2 and in the footnotes of the relevant tables.

**FIGURE 2:**
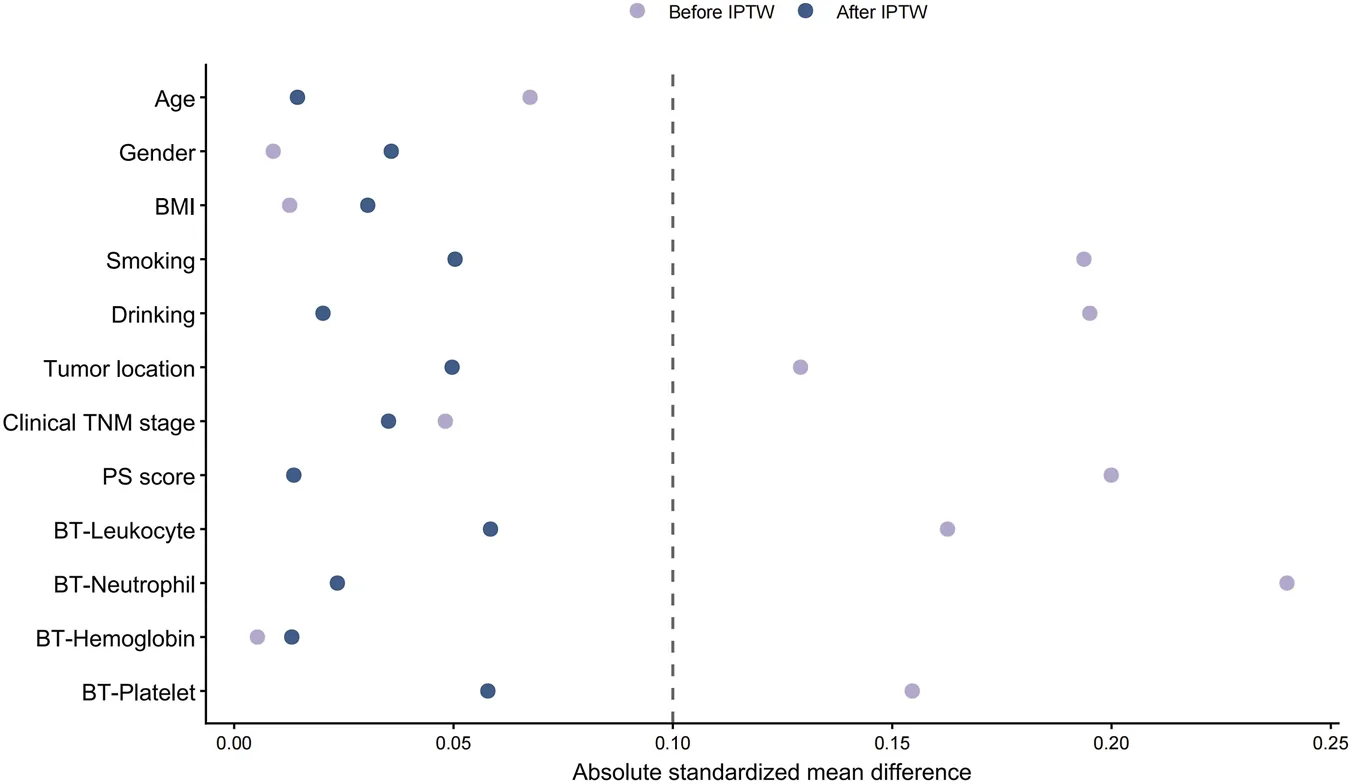
Love plot of absolute standardized mean differences before and after IPTW. For categorical variables with more than two levels, the maximum absolute SMD across all categories is shown. Abbreviations: BMI, body mass index; BT, before treatment; IPTW, inverse probability of treatment weighting; PS, performance status.

Binary outcomes were analyzed using IPTW-weighted Firth-penalized logistic regression to estimate odds ratios (ORs) and 95% confidence intervals (CIs), particularly in the presence of sparse or zero-event data. Weighted risk differences were also reported, with 95% CIs estimated using 2,000 patient-level bootstrap resamples and fixed weights. *P* values were obtained from penalized likelihood-ratio tests and adjusted for multiple comparisons using the Benjamini–Hochberg false discovery rate (FDR) procedure. Covariates that remained imbalanced after weighting were additionally included in the corresponding outcome models.

Eight analysis strategies were compared for the primary hematologic outcomes, comprising the main stabilized IPTW analysis and seven sensitivity analyses evaluating the robustness of the findings to weighting specifications, truncation thresholds, and model assumptions: unadjusted analysis; no weight truncation; truncation at the 5th and 95th percentiles; overlap weighting; IPTW with additional covariate adjustment; restriction to the region of common support; and targeted adjustment for tumor location and baseline platelet count. For each strategy, a Bayesian logistic regression model with weakly informative priors was additionally fitted in parallel. The results are presented in Supplementary Table S3.

Longitudinal blood-cell counts were analyzed using IPTW-weighted generalized estimating equations (GEE) with an exchangeable working correlation structure and robust standard errors. The models included treatment group, time, the group-by-time interaction, the baseline value of the corresponding blood-cell count, and tumor location. Overall effects are presented in Supplementary Table S4; the group main effect represents the adjusted between-group difference at the reference cycle (C1), with cycle-specific estimates and their interaction terms shown therein. Cycle-specific *P* values were adjusted using the FDR procedure. A supplementary sensitivity analysis additionally adjusted for prophylactic G-CSF and is presented in Supplementary Table S5. Categorical outcomes were compared using the Rao–Scott weighted chi-square test.

DFS and OS were estimated using the Kaplan-Meier method, with survival curves, risk tables (weighted effective sample sizes), and hazard ratios all based on IPTW-weighted analyses; between-group comparisons were performed using IPTW-weighted Cox proportional hazards models to estimate hazard ratios (HRs) and 95% CIs; *P* values were derived from likelihood-ratio tests of the weighted Cox models. The proportional hazards assumption was assessed using Schoenfeld residuals (Supplementary Figure S4). Survival analyses were exploratory and were not intended to establish equivalence or noninferiority. Median follow-up was estimated using the reverse Kaplan-Meier method.

Two-sided *P* < 0.05 (or FDR-adjusted *P* < 0.05 where applicable) was considered statistically significant, and adjustment was performed within the corresponding outcome families; OS and DFS were exploratory and not included in the multiplicity-adjustment families. Missing data were not multiply imputed. Binary and survival outcomes were analyzed in their respective analysis sets, and longitudinal counts using available observations under a missing-at-random (MAR) assumption. However, cycle-level missingness was substantial, particularly at later cycles, and mainly reflected patients completing neoadjuvant therapy and proceeding to surgery rather than random missingness; therefore, no sensitivity analysis for departures from MAR was performed, and incomplete-observation bias cannot be excluded. Robust variance estimation was used for weighted models, and ESS assessed information loss after weighting. Given the limited sample size, stratified analyses by treatment heterogeneity could not be performed, but seven sensitivity analyses assessed robustness; supportive care was a post-treatment variable, and no formal mediation analysis was performed. Analyses used R 4.4.2 (primarily with the dplyr, tidyr, survey, logistf, rstanarm, geepack, survival, survminer, ggplot2 packages).

### 2.5 Ethics statement

This study was conducted in accordance with the Declaration of Helsinki. The study was approved by the Ethics Committee of the First Affiliated Hospital of Shantou University Medical College (approval number: B-2023-147). All patients provided written informed consent before enrollment. During the informed consent process, investigators explained the study objectives, procedures, potential risks and benefits, privacy protections, and patient rights. Participants were free to withdraw at any time, and withdrawal did not affect their routine clinical care.

## 3 Results

### 3.1 Patient flow and baseline characteristics

A total of 97 patients were enrolled (69 in the control group and 28 in the trilaciclib group). Nineteen patients had incomplete follow-up (13 controls and 6 trilaciclib-treated patients): 10 transferred to other hospitals, 5 underwent treatment-regimen changes, and 4 were lost to follow-up without post-baseline hematologic data. Data obtained before transfer or regimen change were retained for the relevant analyses (Supplementary Table S1). The four patients without post-baseline data were excluded from all outcome analyses, resulting in a final safety analysis set of 93 patients (65 controls and 28 trilaciclib-treated patients).

Before weighting, imbalances were observed in ECOG performance status, smoking status, alcohol use, tumor location, and baseline blood-cell counts (absolute SMDs ranging from 0.129 to 0.240). After stabilized IPTW, the weighted pseudo-population totaled 92.7 patients (65.1 controls and 27.6 trilaciclib-treated patients), and all covariates achieved an absolute SMD <0.10 (maximum SMD, 0.058; Table 1; Figure 2). The overall ESS was 88.3 (63.3 for the control group and 25.2 for the trilaciclib group). Five patients, all in the control group, fell outside the region of common support, and the maximum weight after truncation was 1.73 (Supplementary Figures S1, S2).

**TABLE 1:** Baseline characteristics before and after stabilized IPTW.

Variables	Before IPTW				After IPTW			
	Total (n = 93)	Control group (n = 65)	Trilaciclib group (n = 28)	SMD	Weighted total (n = 92.7)	Control group (n = 65.1)	Trilaciclib group (n = 27.6)	SMD
Age, n (%)								
<65 years	52 (55.9%)	37 (56.9%)	15 (53.6%)	0.067	51.1 (55.1%)	36.0 (55.3%)	15.1 (54.6%)	0.014
≥65 years	41 (44.1%)	28 (43.1%)	13 (46.4%)		41.6 (44.9%)	29.1 (44.7%)	12.5 (45.4%)	
PS score, n (%)								
0	15 (16.1%)	9 (13.8%)	6 (21.4%)	0.200	15.4 (16.6%)	10.8 (16.5%)	4.6 (16.7%)	0.014
1	71 (76.3%)	51 (78.5%)	20 (71.4%)		70.6 (76.1%)	49.5 (76.1%)	21.0 (76.2%)	
2	7 (7.5%)	5 (7.7%)	2 (7.1%)		6.8 (7.3%)	4.8 (7.4%)	1.9 (7.1%)	
Gender, n (%)								
Female	23 (24.7%)	16 (24.6%)	7 (25.0%)	0.009	23.3 (25.2%)	16.1 (24.7%)	7.3 (26.3%)	0.036
Male	70 (75.3%)	49 (75.4%)	21 (75.0%)		69.4 (74.8%)	49.0 (75.3%)	20.4 (73.7%)	
BMI, n (%)								
<18.5	13 (14.0%)	9 (13.8%)	4 (14.3%)	0.013	13.6 (14.6%)	9.4 (14.4%)	4.2 (15.2%)	0.030
18.5–24	70 (75.3%)	49 (75.4%)	21 (75.0%)		69.0 (74.5%)	48.7 (74.8%)	20.3 (73.5%)	
≥24	10 (10.8%)	7 (10.8%)	3 (10.7%)		10.1 (10.9%)	7.0 (10.8%)	3.1 (11.2%)	
Smoking, n (%)								
Never smoker	37 (39.8%)	24 (36.9%)	13 (46.4%)	0.194	37.7 (40.6%)	26.0 (39.9%)	11.7 (42.4%)	0.050
Ever smoker	56 (60.2%)	41 (63.1%)	15 (53.6%)		55.0 (59.4%)	39.1 (60.1%)	15.9 (57.6%)	
Drinking, n (%)								
Never drinker	64 (68.8%)	43 (66.2%)	21 (75.0%)	0.195	64.3 (69.4%)	45.0 (69.1%)	19.3 (70.0%)	0.020
Ever drinker	29 (31.2%)	22 (33.8%)	7 (25.0%)		28.4 (30.6%)	20.1 (30.9%)	8.3 (30.0%)	
Tumor location, n (%)								
Lower	29 (31.2%)	20 (30.8%)	9 (32.1%)	0.129	27.9 (30.1%)	20.0 (30.8%)	7.9 (28.5%)	0.050
Middle	62 (66.7%)	44 (67.7%)	18 (64.3%)		62.4 (67.3%)	43.4 (66.6%)	19.0 (68.8%)	
Upper	2 (2.2%)	1 (1.5%)	1 (3.6%)		2.4 (2.6%)	1.7 (2.6%)	0.7 (2.7%)	
Clinical TNM stage, n (%)								
Stage II	19 (20.4%)	12 (18.5%)	7 (25.0%)	0.048	19.5 (21.1%)	13.3 (20.4%)	6.2 (22.5%)	0.035
Stage III	47 (50.5%)	32 (49.2%)	15 (53.6%)		46.1 (49.7%)	32.6 (50.1%)	13.5 (50.5%)	
Stage IVA	27 (29.0%)	21 (32.3%)	6 (21.4%)		27.1 (29.3%)	19.2 (29.6%)	8.0 (30.0%)	
BT-leukocyte, median (Q1, Q3)	7.52(6.47, 8.64)	7.34(6.40, 8.64)	7.58(6.49, 8.46)	0.163	7.47(6.40, 8.64)	7.30(6.35, 8.64)	7.59(6.47, 8.47)	0.058
BT-neutrophil, median (Q1, Q3)	4.66(4.02, 5.95)	4.69(4.05, 5.95)	4.52(3.91, 5.58)	0.240	4.66(4.02, 5.95)	4.66(4.00, 5.94)	4.74(4.09, 6.33)	0.024
BT-hemoglobin, median (Q1, Q3)	130.0(121.0, 140.0)	130.0(121.0, 138.0)	128.5(121.8, 140.0)	0.005	130.0(122.0, 140.0)	130.0(122.0, 138.0)	130.0(122.0, 140.0)	0.013
BT-platelet, median (Q1, Q3)	264.0(229.0, 315.0)	257.0(221.0, 308.0)	279.5(248.0, 317.2)	0.155	264.0(224.0, 315.0)	260.0(218.0, 321.0)	266.0(241.0, 315.0)	0.058

Continuous variables are median (Q1, Q3); categorical variables are n (%). Stabilized IPTW with 1st/99th percentile truncation was used for baseline balance, assessed by absolute SMD (<0.10 acceptable). ESS after IPTW: total = 88.3, control = 63.3, trilaciclib = 25.2 (calculated as (άw)^2^/άw^2^; values are cohort‐specific and not additive). Units: BT‐leukocyte and BT‐neutrophil, ×10^9^ cells/L; BT‐hemoglobin, g/L; BT‐platelet, ×10^9^/L. Abbreviations: BMI, body mass index; BT, before treatment; ESS, effective sample size; IPTW, inverse probability of treatment weighting; PS, performance status; SMD, standardized mean difference.

Among the 93 patients in the safety analysis set, 75.3% were male, 44.1% were aged ≥65 years, 60.2% had a history of smoking, and 66.7% had tumors located in the middle thoracic esophagus (Table 1). The survival analysis included 78 patients with evaluable OS and DFS data; the median follow-up was 21.0 months (IQR, 15.0–23.3) in the control group and 20.7 months (IQR, 14.2–22.0) in the trilaciclib group.

### 3.2 Grade 3–4 hematologic adverse events and supportive care use in the overall cohort

Hematologic outcomes are summarized in Table 2 and Figure 3A. The weighted incidence of grade 3–4 CIM was lower in the trilaciclib group than in the control group (3.0% vs. 31.2%; crude events, 1/28 vs. 19/65; OR, 0.11; 95% CI, 0.01–0.48; FDR-adjusted *P* = 0.007). Grade 3–4 chemotherapy-induced neutropenia (CIN) was also lower in the trilaciclib group (3.0% vs. 28.3%; OR, 0.12; 95% CI, 0.01–0.55; FDR-adjusted *P* = 0.010), as was grade 3–4 chemotherapy-induced anemia (CIA; 0.0% vs. 17.3%; OR, 0.08; 95% CI, <0.01–0.67; FDR-adjusted *P* = 0.030). No grade 3–4 chemotherapy-induced thrombocytopenia (CIT) events were observed in the trilaciclib group (0/28), compared with 3/65 in the control group (weighted incidences, 0.0% vs. 4.7%); the difference was not statistically significant. Grade 3–4 CIT, severe neutropenia (SN), and febrile neutropenia (FN) did not differ significantly between groups (FN: weighted incidence, 3.0% vs. 10.2%; crude events, 1/28 vs. 6/65). Sensitivity analyses were consistent with the primary results (Supplementary Table S3).

**TABLE 2:** IPTW-weighted analysis of grade 3–4 hematologic adverse events and supportive care in the overall cohort.

Outcome	Events, n/N (control)	Events, n/N (trilaciclib)	Weighted rate, % (control)	Weighted rate, % (trilaciclib)	RD, % (95% CI)	OR (95% CI)	*P* value	*P* (FDR-adjusted)
Grade 3–4 CIM	19/65	1/28	31.2	3.0	−28.2 (−40.4, −14.6)	0.11 (0.01–0.48)	0.002	0.007
Grade 3–4 CIN	17/65	1/28	28.3	3.0	−25.3 (−37.3, −12.1)	0.12 (0.01–0.55)	0.004	0.010
Grade 3–4 CIA	11/65	0/28	17.3	0.0	−17.3 (−26.7, −9.1)	0.08 (<0.01–0.67)	0.014	0.030
Grade 3–4 CIT	3/65	0/28	4.7	0.0	−4.7 (−10.6, 0.0)	0.31 (<0.01–3.33)	0.382	0.414
Severe neutropenia	8/65	1/28	14.1	3.0	−11.1 (−21.8, 0.2)	0.28 (0.02–1.40)	0.133	0.193
Febrile neutropenia	6/65	1/28	10.2	3.0	−7.2 (−16.9, 3.0)	0.40 (0.03–2.10)	0.307	0.362
Treatment delay	8/65	3/28	13.4	8.3	−5.2 (−17.6, 8.1)	0.66 (0.13–2.47)	0.557	0.557
Total G-CSF use	52/65	10/28	79.7	34.4	−45.3 (−66.2, −23.1)	0.14 (0.05–0.36)	<0.001	<0.001
Prophylactic G-CSF use	44/65	8/28	66.8	28.3	−38.5 (−59.7, −15.8)	0.21 (0.08–0.52)	<0.001	0.004
Therapeutic G-CSF use	18/65	3/28	28.7	8.6	−20.1 (−33.9, −5.2)	0.27 (0.06–0.91)	0.034	0.061
TPO use	20/65	3/28	30.6	10.4	−20.3 (−34.7, −3.9)	0.30 (0.07–0.94)	0.038	0.061
ESA use	19/65	1/28	29.6	3.7	−25.9 (−38.2, −11.1)	0.13 (0.01–0.56)	0.004	0.010
Blood transfusion	8/65	1/28	12.6	3.0	−9.6 (−20.1, 1.8)	0.32 (0.03–1.61)	0.184	0.239

Stabilized IPTW, was used; weights were truncated at the 1st/99th percentiles, and all |SMD| <0.10 after weighting. Outcomes were analyzed as binary (Grade 0–2 vs. 3–4 for hematologic adverse events; yes/no for others). Unweighted n/N are shown. Weighted rates, risk differences (bootstrap 95% CIs), and odds ratios (IPTW-weighted Firth-penalized logistic regression) are reported; P values are penalized likelihood-ratio tests with Benjamini-Hochberg FDR adjustment. Given the small sample size, results are exploratory and hypothesis-generating. Effective sample sizes: 63.3 (control) and 25.2 (trilaciclib). Abbreviations: CIM, chemotherapy-induced myelosuppression; CIN, chemotherapy-induced neutropenia; CIA, chemotherapy-induced anemia; CIT, chemotherapy-induced thrombocytopenia; SN, severe neutropenia; FN, febrile neutropenia; G-CSF, granulocyte colony-stimulating factor; TPO, thrombopoietin; ESA, erythropoiesis-stimulating agent; IPTW, inverse probability of treatment weighting; RD, risk difference; OR, odds ratio; FDR, false discovery rate.

**FIGURE 3:**
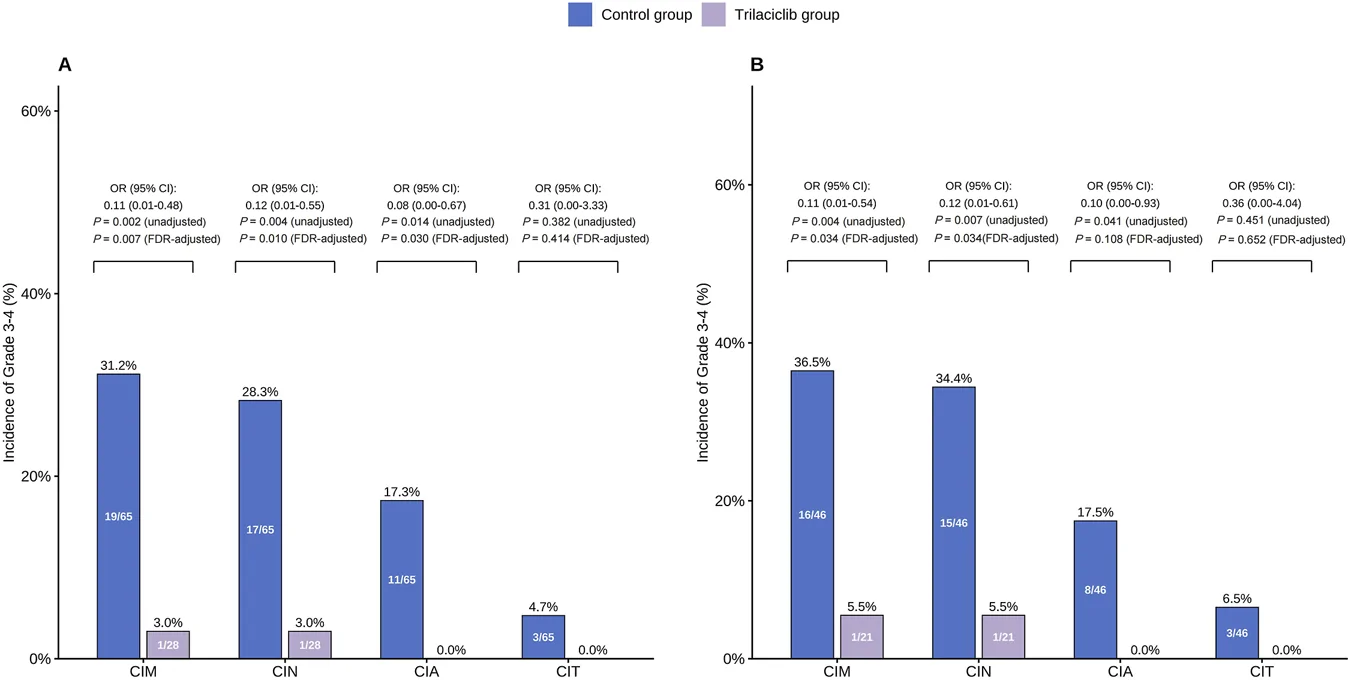
Grade 3–4 hematologic adverse events in the perioperative cohort **(A)** and the nICT (neoadjuvant immunochemotherapy) subgroup **(B)**. Bars show IPTW-weighted incidence (%), and numbers inside bars are raw event counts (n/N). Odds ratios (ORs) and 95% confidence intervals (CIs) were derived from Firth-penalized weighted logistic regression to account for zero-event outcomes. The CIs correspond to the unadjusted models. P values are presented as both unadjusted and FDR-adjusted (Benjamini–Hochberg procedure applied across the four AE categories within each cohort separately) to address multiple comparisons. Because individual AE categories are not mutually exclusive, totals should not be summed across categories. Because of the small number of events, the CIs for some endpoints (CIA, CIT) are wide and should be interpreted with caution. Abbreviations: CIM, chemotherapy-induced myelosuppression; CIN, chemotherapy-induced neutropenia; CIA, chemotherapy-induced anemia; CIT, chemotherapy-induced thrombocytopenia; IPTW, inverse probability of treatment weighting; FDR, false discovery rate; OR, odds ratio; CI, confidence interval; nICT, neoadjuvant immunochemotherapy.

Use of total G-CSF was lower in the trilaciclib group (34.4% vs. 79.7%; OR, 0.14; 95% CI, 0.05–0.36; FDR-adjusted *P* < 0.001), as was prophylactic G-CSF use (28.3% vs. 66.8%; OR, 0.21; 95% CI, 0.08–0.52; FDR-adjusted *P* = 0.004) and ESA use (3.7% vs. 29.6%; OR, 0.13; 95% CI, 0.01–0.56; FDR-adjusted *P* = 0.010). Differences in therapeutic G-CSF use (unadjusted *P* = 0.034) and TPO use (unadjusted *P* = 0.038) were no longer statistically significant after FDR adjustment (both FDR-adjusted *P* = 0.061). Transfusion use and treatment delay attributable to hematologic toxicity did not differ significantly between groups.

### 3.3 Grade 3–4 hematologic adverse events and supportive care use in the nICT subgroup

Results for the nICT subgroup are presented in Table 3 and Figure 3B. The subgroup was reweighted using the simplified five-covariate propensity-score model (ESS: 43.7 for the control group and 17.9 for the trilaciclib group). Smoking status, tumor location, and clinical TNM stage remained imbalanced after weighting and were therefore additionally adjusted in the outcome models (Supplementary Figure S3). The weighted incidence of grade 3–4 CIM was lower in the trilaciclib group (5.5% vs. 36.5%; crude events, 1/21 vs. 16/46; OR, 0.11; 95% CI, 0.01–0.54; FDR-adjusted *P* = 0.034), as was grade 3–4 CIN (5.5% vs. 34.4%; OR, 0.12; 95% CI, 0.01–0.61; FDR-adjusted *P* = 0.034). The difference in grade 3–4 CIA was not statistically significant after FDR adjustment (unadjusted *P* = 0.041; FDR-adjusted *P* = 0.108). ESA use was lower in the trilaciclib group (0.0% vs. 23.9%; OR, 0.06; 95% CI, <0.01–0.56; FDR-adjusted *P* = 0.034). The difference in total G-CSF use was not statistically significant after FDR adjustment (38.7% vs. 70.6%; unadjusted *P* = 0.034; FDR-adjusted *P* = 0.108). No statistically significant between-group differences were observed for the remaining prespecified endpoints.

**TABLE 3:** IPTW-weighted analysis of grade 3–4 hematologic adverse events and supportive care in the nICT subgroup.

Outcome	Events, n/N (control)	Events, n/N (trilaciclib)	Weighted rate, % (control)	Weighted rate, % (trilaciclib)	RD, % (95% CI)	OR (95% CI)	*P* value	*P* (FDR-adjusted)
Grade 3–4 CIM	16/46	1/21	36.5	5.5	−31.0 (−47.5, −13.0)	0.11 (0.01–0.54)	0.004	0.034
Grade 3–4 CIN	15/46	1/21	34.4	5.5	−28.9 (−45.1, −10.7)	0.12 (0.01–0.61)	0.007	0.034
Grade 3–4 CIA	8/46	0/21	17.5	0.0	−17.5 (−30.7, −6.7)	0.10 (<0.01–0.93)	0.041	0.108
Grade 3–4 CIT	3/46	0/21	6.5	0.0	−6.5 (−14.3, 0.0)	0.36 (<0.01–4.04)	0.451	0.652
Severe neutropenia	6/46	1/21	14.5	5.5	−9.0 (−22.0, 7.7)	0.27 (0.02–1.68)	0.178	0.289
Febrile neutropenia	4/46	1/21	9.9	5.5	−4.4 (−16.0, 11.4)	0.54 (0.04–3.31)	0.532	0.674
Treatment delay	5/46	3/21	11.0	12.4	1.4 (−15.8, 17.7)	1.00 (0.17–5.02)	0.997	0.997
Total G-CSF use	33/46	8/21	70.6	38.7	−31.9 (−57.3, −6.2)	0.33 (0.11–0.92)	0.034	0.108
Prophylactic G-CSF use	26/46	6/21	54.0	27.8	−26.3 (−52.8, −1.6)	0.36 (0.11–1.04)	0.059	0.128
Therapeutic G-CSF use	11/46	3/21	25.1	17.8	−7.4 (−28.9, 16.2)	0.70 (0.18–2.33)	0.570	0.674
TPO use	14/46	2/21	29.3	12.6	−16.7 (−37.8, 6.9)	0.41 (0.09–1.43)	0.167	0.289
ESA use	11/46	0/21	23.9	0.0	−23.9 (−38.2, −11.7)	0.06 (<0.01–0.56)	0.008	0.034
Blood transfusion	5/46	1/21	10.4	5.5	−4.9 (−17.7, 10.2)	0.67 (0.07–3.52)	0.652	0.706

Stabilized IPTW was re-estimated in the nICT subgroup using a simplified PS, model; weights were truncated at the 1st/99th percentiles. Covariates remaining imbalanced after IPTW (smoking, tumor location, clinical TNM, stage) were additionally adjusted in the Firth-penalized logistic regression (doubly robust approach). Outcomes were analyzed as binary (Grade 0–2 vs. 3–4 for hematologic adverse events; yes/no for supportive care). Unweighted n/N are shown; weighted rates, risk differences (bootstrap 95% CIs), and odds ratios (IPTW-weighted Firth-penalized logistic regression) are reported. P values are penalized likelihood-ratio tests with Benjamini-Hochberg FDR, adjustment. Given the small sample size, results are exploratory and hypothesis-generating. Effective sample sizes were 43.7 for the control group and 17.9 for the trilaciclib group. Abbreviations: CIM, chemotherapy-induced myelosuppression; CIN, chemotherapy-induced neutropenia; CIA, chemotherapy-induced anemia; CIT, chemotherapy-induced thrombocytopenia; SN, severe neutropenia; FN, febrile neutropenia; G-CSF, granulocyte colony-stimulating factor; TPO, thrombopoietin; ESA, erythropoiesis-stimulating agent; nICT, neoadjuvant immunochemotherapy; IPTW, inverse probability of treatment weighting; RD, risk difference; OR, odds ratio; FDR, false discovery rate.

### 3.4 Longitudinal changes in hematologic parameters

The number of patients contributing data declined at cycle four in both groups, primarily because patients who completed neoadjuvant therapy proceeded to surgery before additional cycles were administered; the numbers of patients contributing data at each cycle are shown in Figure 4.

**FIGURE 4:**
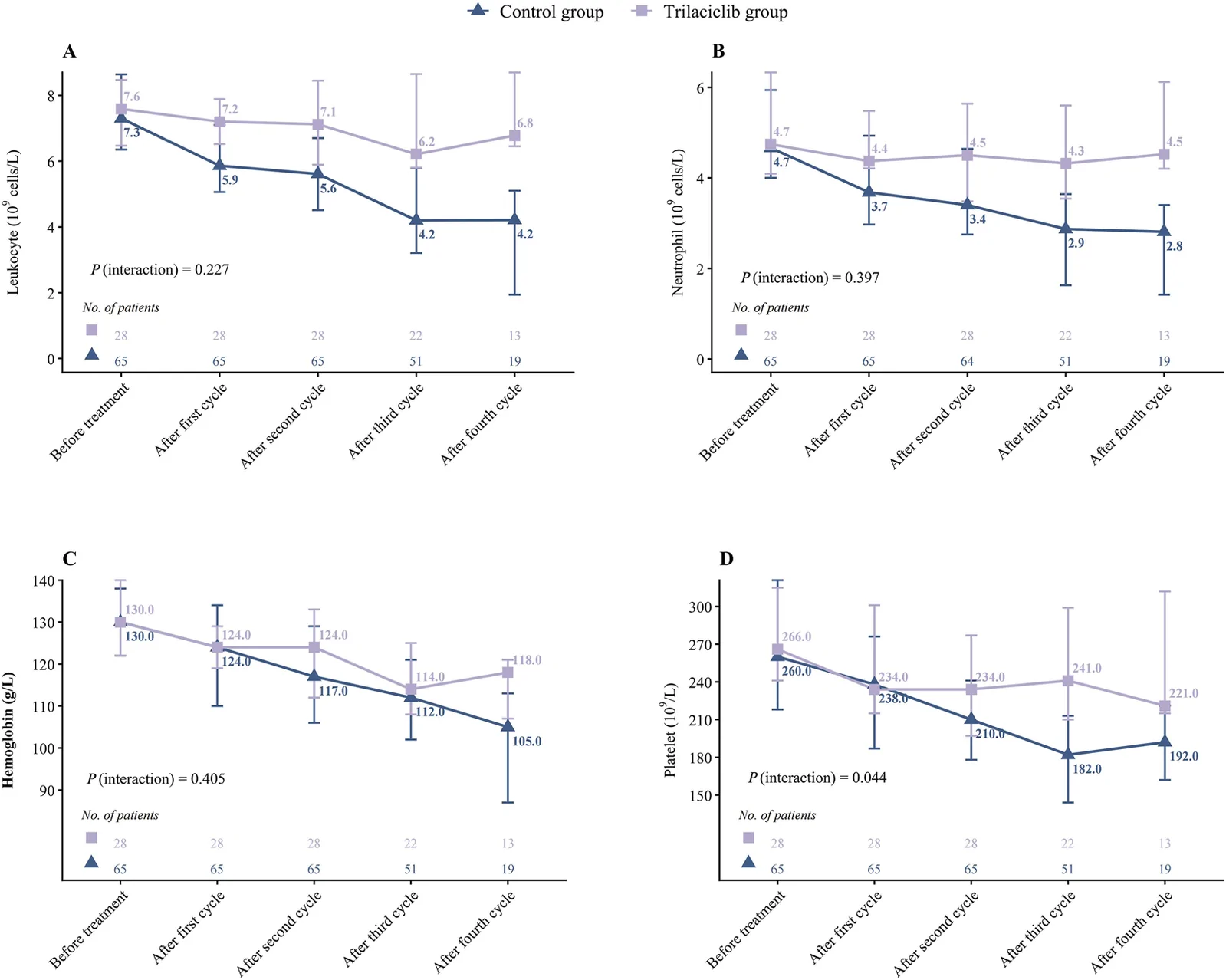
PTW-weighted hematological parameter changes in control vs. trilaciclib groups across treatment cycles. **(A)** Leukocyte; **(B)** Neutrophil; **(C)** Hemoglobin; **(D)** Platelet. Data are IPTW-weighted median (IQR). Stabilized IPTW was estimated using a propensity score model including 12 baseline covariates; weights were truncated at the 1st and 99th percentiles. P values for the treatment-by-time interaction were derived from weighted generalized estimating equations (GEE) models with FDR adjustment (Supplementary Table S4). The decline in patient numbers at cycle 4 reflects the completion of the planned neoadjuvant cycles and progression to surgery, rather than discontinuation solely due to hematologic toxicity. Numbers below each panel indicate the number of patients with available data at each time point (blue triangles, Control; purple squares, Trilaciclib). Abbreviations: IPTW, inverse probability of treatment weighting; IQR, interquartile range; FDR, false discovery rate; GEE, generalized estimating equations.

In the overall cohort, the main effects of time were statistically significant for all four hematologic parameters (all FDR-adjusted *P* < 0.001; Supplementary Table S4). A statistically significant group-by-time interaction was observed for platelet counts only (FDR-adjusted *P* = 0.044). In the sensitivity analysis additionally adjusting for prophylactic G-CSF use in this cohort, the treatment main effects on leukocyte and neutrophil counts reached statistical significance (Wald *P* = 0.018 and 0.046, respectively), and the platelet group-by-time interaction remained statistically significant (Wald *P* = 0.029; Supplementary Table S5).

In cycle-specific analyses, weighted adjusted leukocyte counts were higher in the trilaciclib group at cycles 2–4 (adjusted mean difference, 1.32–2.41 × 10^9^/L; all FDR-adjusted *P* ≤ 0.004). Neutrophil counts were higher at cycles 2 and 3 (FDR-adjusted *P* = 0.009 and 0.002, respectively), and platelet counts were higher at cycles 3 and 4 (FDR-adjusted *P* = 0.002 and 0.009, respectively). Hemoglobin did not differ significantly at any cycle.

In the nICT subgroup, the main effect of treatment group on leukocyte counts was statistically significant (FDR-adjusted *P* = 0.015), with higher counts in the trilaciclib group at cycles 1–4 (all cycle-specific FDR-adjusted *P* ≤ 0.020). Neutrophil counts were higher at cycles 2–4, and hemoglobin and platelet counts were higher at cycle 3 (all cycle-specific FDR-adjusted *P* ≤ 0.020). No group-by-time interactions were statistically significant in the nICT subgroup (Table 4; Figure 5).

**TABLE 4:** IPTW-weighted longitudinal analysis of hematologic parameters in the nICT subgroup.

Variables	Time	Raw n (control/trilaciclib)	Weighted median (IQR) (control)	Weighted median (IQR) (trilaciclib)	Adjusted mean difference (95% CI)	*P* (FDR-adjusted)
Leukocyte	C1	46/21	6.12 (5.06, 7.10)	7.21 (6.21, 8.42)	1.26 (0.30–2.21)	0.010 (0.020)
	C2	46/21	5.66 (4.72, 6.42)	7.25 (5.77, 8.45)	1.41 (0.66–2.15)	<0.001 (0.003)
	C3	36/16	4.80 (3.47, 5.79)	6.16 (5.80, 6.41)	1.40 (0.43–2.36)	0.004 (0.018)
	C4	11/9	4.21 (1.94, 4.82)	6.54 (6.45, 7.21)	2.56 (1.03–4.09)	0.001 (0.006)
Neutrophil	C1	46/21	3.75 (3.12, 4.93)	4.25 (3.98, 5.83)	0.84 (0.05–1.64)	0.038 (0.061)
	C2	45/21	3.41 (2.87, 4.39)	4.60 (3.54, 6.42)	1.20 (0.51–1.89)	<0.001 (0.006)
	C3	36/16	3.14 (1.86, 4.10)	3.79 (3.54, 4.32)	1.04 (0.25–1.83)	0.010 (0.020)
	C4	11/9	2.56 (1.42, 3.30)	4.52 (4.12, 4.68)	1.50 (0.38–2.62)	0.009 (0.020)
Hemoglobin	C1	46/21	123.00 (108.00, 132.00)	124.00 (115.00, 131.00)	1.34 (−3.70–6.38)	0.602 (0.656)
	C2	46/21	117.00 (106.00, 129.00)	124.00 (106.00, 127.00)	8.39 (−5.99–22.77)	0.253 (0.337)
	C3	36/16	112.00 (104.00, 121.00)	117.00 (110.00, 134.00)	9.92 (2.25–17.60)	0.011 (0.020)
	C4	11/9	105.00 (87.00, 113.00)	108.00 (101.00, 121.00)	8.06 (−5.27–21.38)	0.236 (0.337)
Platelet	C1	46/21	245.00 (187.00, 279.00)	248.00 (210.00, 301.00)	5.31 (−23.97–34.60)	0.722 (0.722)
	C2	46/21	214.00 (186.00, 252.00)	257.00 (197.00, 278.00)	9.65 (−27.97–47.27)	0.615 (0.656)
	C3	36/16	197.00 (157.00, 214.00)	241.00 (214.00, 281.00)	49.39 (14.27–84.51)	0.006 (0.019)
	C4	11/9	192.00 (162.00, 241.00)	215.00 (212.00, 312.00)	45.41 (−38.50–129.31)	0.289 (0.355)

IPTW-weighted longitudinal analysis of hematologic parameters in the nICT subgroup (Trilaciclib vs. Control). Data are IPTW-weighted estimates from a pseudo-population, not actual patient counts. Time points C1–C4 correspond to treatment cycles 1–4, respectively. Raw n indicates the actual number of patients with available data at each cycle. Adjusted mean differences (95% CIs) at each cycle were derived from weighted GEE models adjusting for the covariates described in the Methods. FDR-adjusted P values are shown for cycle-specific comparisons (Benjamini-Hochberg procedure applied across the four time points within each marker). Effective sample sizes were 43.7 for the control group and 17.9 for the trilaciclib group. Overall GEE effects are reported in Supplementary Table S4. Units: leukocyte and neutrophil counts, ×109 cells/L; hemoglobin, g/L; platelet count, ×109/L. Because of the small sample size, results should be interpreted as exploratory. Abbreviations: IPTW, inverse probability of treatment weighting; GEE, generalized estimating equations; IQR, interquartile range; FDR, false discovery rate; CI, confidence interval.

**FIGURE 5:**
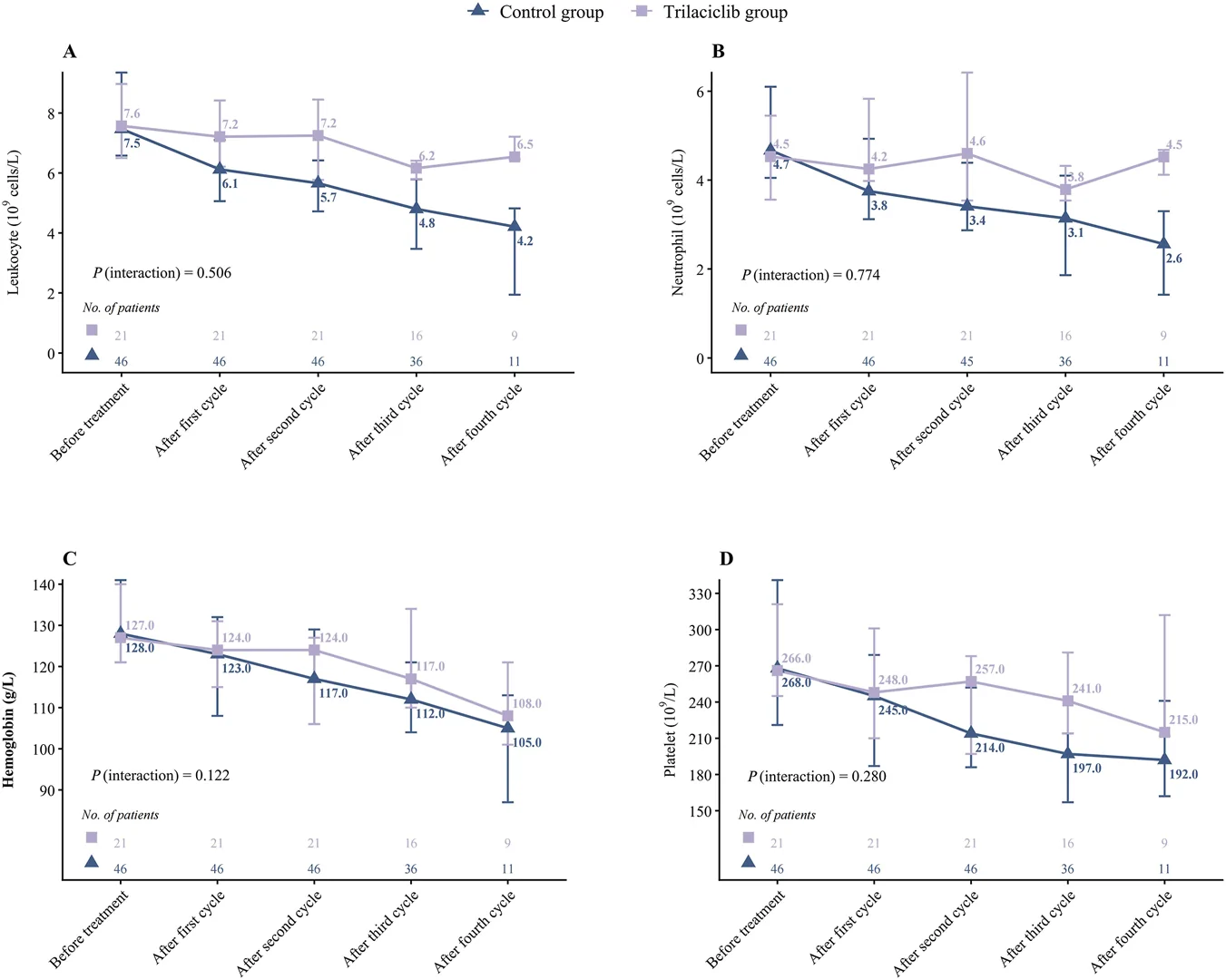
IPTW-weighted hematological parameter changes in the nICT subgroup (control vs. trilaciclib). **(A)** Leukocyte; **(B)** Neutrophil; **(C)** Hemoglobin; **(D)** Platelet. Data are IPTW-weighted median (IQR). Stabilized IPTW was estimated using a simplified propensity score model including age, PS score, clinical TNM stage, tumor location, and baseline platelet count; weights were truncated at the 1st and 99th percentiles. P values for the treatment-by-time interaction were derived from weighted generalized estimating equations (GEE) models with FDR adjustment (Supplementary Table S4). The decline in patient numbers at cycle 4 reflects the completion of the planned neoadjuvant cycles and progression to surgery, rather than discontinuation solely due to hematologic toxicity. Numbers below each panel indicate the number of patients with available data at each time point (blue triangles, Control; purple squares, Trilaciclib). Abbreviations: IPTW, inverse probability of treatment weighting; IQR, interquartile range; GEE, generalized estimating equations; nICT, neoadjuvant immunochemotherapy.

### 3.5 Nonhematologic adverse events

Nonhematologic adverse events are summarized in Table 5. The incidences of decreased appetite, vomiting, diarrhea, constipation, fatigue, abnormal liver function tests, abnormal renal function tests, and electrolyte disturbances did not differ significantly between groups (Rao–Scott weighted chi-square test). Most events were grade 1–2. Grade 3 events were observed only for liver function abnormalities, with weighted incidences of 10.2% in the control group and 6.0% in the trilaciclib group; the difference was not statistically significant. No grade 4 or higher nonhematologic events were observed. The most common grade 2 events were decreased appetite and vomiting, with weighted overall incidences of 17.3% and 13.6%, respectively.

**TABLE 5:** IPTW-weighted non-hematologic adverse events in the perioperative cohort and the nICT subgroup.

Variables	Perioperative cohort				nICT			
	Weighted total(n = 92.7)	Control group(n = 65.1)	Trilaciclib group(n = 27.6)	*P* value	Weighted total(n = 66.4)	Control group(n = 46.1)	Trilaciclib group(n = 20.2)	*P* value
Decreased appetite								
Grade 0–1	76.7 (82.72)	53.6 (82.28)	23.1 (83.76)	0.875	56.4 (84.97)	38.5 (83.54)	17.9 (88.23)	0.653
Grade 2	16.0 (17.28)	11.5 (17.72)	4.5 (16.24)		10.0 (15.03)	7.6 (16.46)	2.4 (11.77)	
Vomiting								
Grade 0–1	80.1 (86.41)	56.1 (86.22)	24.0 (86.87)	0.934	56.5 (85.20)	38.7 (83.90)	17.8 (88.14)	0.685
Grade 2	12.6 (13.59)	9.0 (13.78)	3.6 (13.13)		9.8 (14.80)	7.4 (16.10)	2.4 (11.86)	
Diarrhea								
Grade 0–1	72.9 (94.10)	50.6 (93.05)	22.3 (96.59)	0.511	47.5 (95.49)	33.2 (95.42)	14.3 (95.64)	0.969
Grade 2	4.6 (5.90)	3.8 (6.95)	0.8 (3.41)		2.2 (4.51)	1.6 (4.58)	0.7 (4.36)	
Constipation								
Grade 0–1	90.3 (97.38)	63.4 (97.33)	26.9 (97.50)	0.957	65.6 (98.81)	46.1 (100.00)	19.5 (96.11)	0.140
Grade 2	2.4 (2.62)	1.7 (2.67)	0.7 (2.50)		0.8 (1.19)	0.0 (0.00)	0.8 (3.89)	
Fatigue								
Grade 0–1	83.4 (89.90)	57.8 (88.74)	25.6 (92.63)	0.591	61.8 (93.06)	42.2 (91.43)	19.6 (96.77)	0.361
Grade 2	9.4 (10.10)	7.3 (11.26)	2.0 (7.37)		4.6 (6.94)	4.0 (8.57)	0.7 (3.23)	
Hepatic function abnormal								
Grade 0–1	66.0 (71.64)	45.9 (70.45)	20.1 (74.50)	0.808	51.1 (78.14)	34.5 (74.77)	16.6 (86.21)	0.419
Grade 2	17.8 (19.37)	12.6 (19.31)	5.3 (19.53)		9.5 (14.46)	7.3 (15.83)	2.2 (11.21)	
Grade 3	8.3 (8.99)	6.7 (10.24)	1.6 (5.97)		4.8 (7.39)	4.3 (9.40)	0.5 (2.59)	
Renal function abnormal								
Grade 0–1	85.2 (91.93)	60.6 (93.10)	24.6 (89.16)	0.549	62.3 (93.93)	43.3 (93.79)	19.1 (94.23)	0.936
Grade 2	7.5 (8.07)	4.5 (6.90)	3.0 (10.84)		4.0 (6.07)	2.9 (6.21)	1.2 (5.77)	
Hyperkalemia								
Grade 0–1	87.5 (94.32)	62.0 (95.18)	25.5 (92.31)	0.620	62.0 (93.37)	43.4 (94.09)	18.6 (91.74)	0.722
Grade 2	5.3 (5.68)	3.1 (4.82)	2.1 (7.69)		4.4 (6.63)	2.7 (5.91)	1.7 (8.26)	
Hypokalemia								
Grade 0–1	82.7 (89.19)	57.0 (87.56)	25.7 (93.03)	0.461	62.2 (93.81)	42.5 (92.17)	19.7 (97.54)	0.275
Grade 2	10.0 (10.81)	8.1 (12.44)	1.9 (6.97)		4.1 (6.19)	3.6 (7.83)	0.5 (2.46)	
Hyponatremia								
Grade 0–1	82.3 (88.79)	56.6 (86.99)	25.7 (93.03)	0.425	62.9 (94.80)	43.2 (93.60)	19.7 (97.54)	0.392
Grade 2	10.4 (11.21)	8.5 (13.01)	1.9 (6.97)		3.4 (5.20)	2.9 (6.40)	0.5 (2.46)	
Hypocalcemia								
Grade 0–1	86.8 (93.63)	60.1 (92.24)	26.8 (96.90)	0.378	63.5 (95.72)	44.2 (95.78)	19.4 (95.59)	0.972
Grade 2	5.9 (6.37)	5.1 (7.76)	0.9 (3.10)		2.8 (4.28)	1.9 (4.22)	0.9 (4.41)	

Stabilized IPTW was used to balance baseline characteristics; weights were truncated at the 1st and 99th percentiles. For the perioperative cohort, a full propensity score model with 12 baseline covariates was used; for the nICT subgroup, a simplified model with 5 covariates was used. Data are presented as IPTW-weighted pseudo-counts (weighted percentage) for the adjusted pseudo-population and are not actual patient counts. P values are from Rao-Scott weighted chi-square tests. ESS after IPTW: perioperative cohort, 88.3 (total), 63.3 (control), and 25.2 (trilaciclib); nICT subgroup, 61.5 (total), 43.7 (control), and 17.9 (trilaciclib). ESS was calculated as (Σw)^2^/Σw^2^. Grade 3 or Grade 4 events that did not occur are omitted; missing grades indicate zero events. Abbreviations: ESS, effective sample size; IPTW, inverse probability of treatment weighting; nICT, neoadjuvant immunochemotherapy.

### 3.6 Pathologic outcomes in the nICT subgroup

Pathologic outcomes are presented in Table 6. Among the 67 patients in the nICT subgroup, 52 had evaluable surgical specimens (37 controls and 15 trilaciclib-treated patients); the remaining 15 patients had no evaluable specimens owing to transfer to another hospital or treatment-regimen change and were therefore excluded from this analysis.

**TABLE 6:** Pathological outcomes in the nICT subgroup.

Outcome	N (available/total)	Events, n/N (control)	Events, n/N (trilaciclib)	OR (95% CI)	*P* value	*P* (FDR-adjusted)
pCR	52/52	8/37	4/15	1.67 (0.39–7.00)	0.481	0.779
MPR	52/52	19/37	8/15	0.72 (0.20–2.46)	0.602	0.779
ypT0-2 (vs. ypT3-4)	52/52	24/37	10/15	1.05 (0.28–4.42)	0.941	0.941
ypN0 (vs. ypN+)	52/52	21/37	9/15	0.69 (0.18–2.58)	0.583	0.779
ypTNM I-II (vs. III-IVA)	52/52	21/37	9/15	0.69 (0.18–2.58)	0.583	0.779
R0 resection	52/52	35/37	15/15	1.88 (0.13–256.06)	0.668	0.779

Data are presented as unweighted counts (n/N). Complete-case analysis included 52 patients; 15 patients were excluded because 10 were transferred to other institutions and five underwent regimen modifications. Pathological assessments were performed by pathologists blinded to treatment allocation. ORs and 95% CIs were estimated using IPTW-weighted Firth-penalized logistic regression. *P* values were adjusted for multiple comparisons using the Benjamini-Hochberg false discovery rate procedure. Because no unadjusted P value was nominally significant, the adjusted P values fell within a narrow range (0.779–0.941). pCR was included in the MPR category. This exploratory analysis was underpowered and was not designed to establish non-inferiority. Abbreviations: CI, confidence interval; FDR, false discovery rate; IPTW, inverse probability of treatment weighting; MPR, major pathological response; OR, odds ratio; pCR, pathological complete response; ypTNM, post-treatment pathological tumor-node-metastasis stage.

The pCR rate was 26.7% (4/15) in the trilaciclib group and 21.6% (8/37) in the control group (OR, 1.67; 95% CI, 0.39–7.00; FDR-adjusted *P* = 0.779). The MPR rate, including pCR, was 53.3% versus 51.4% (OR, 0.72; 95% CI, 0.20–2.46; FDR-adjusted *P* = 0.779). The R0 resection rate was 100% versus 94.6% (OR, 1.88; 95% CI, 0.13–256.06; FDR-adjusted *P* = 0.779). The distributions of ypT, ypN, and ypTNM stage did not differ significantly between groups.

### 3.7 Exploratory survival outcomes

Weighted Kaplan–Meier curves, numbers at risk, and HR estimates are shown in Figure 6. In the overall cohort, 8 deaths and 14 DFS events (recurrence, progression, or death) occurred in the control group, compared with 2 deaths and 5 DFS events in the trilaciclib group. OS did not differ significantly between groups (HR, 0.979; 95% CI, 0.210–4.568; *P* = 0.976), nor did DFS (HR, 0.916; 95% CI, 0.309–2.712; *P* = 0.866).

**FIGURE 6:**
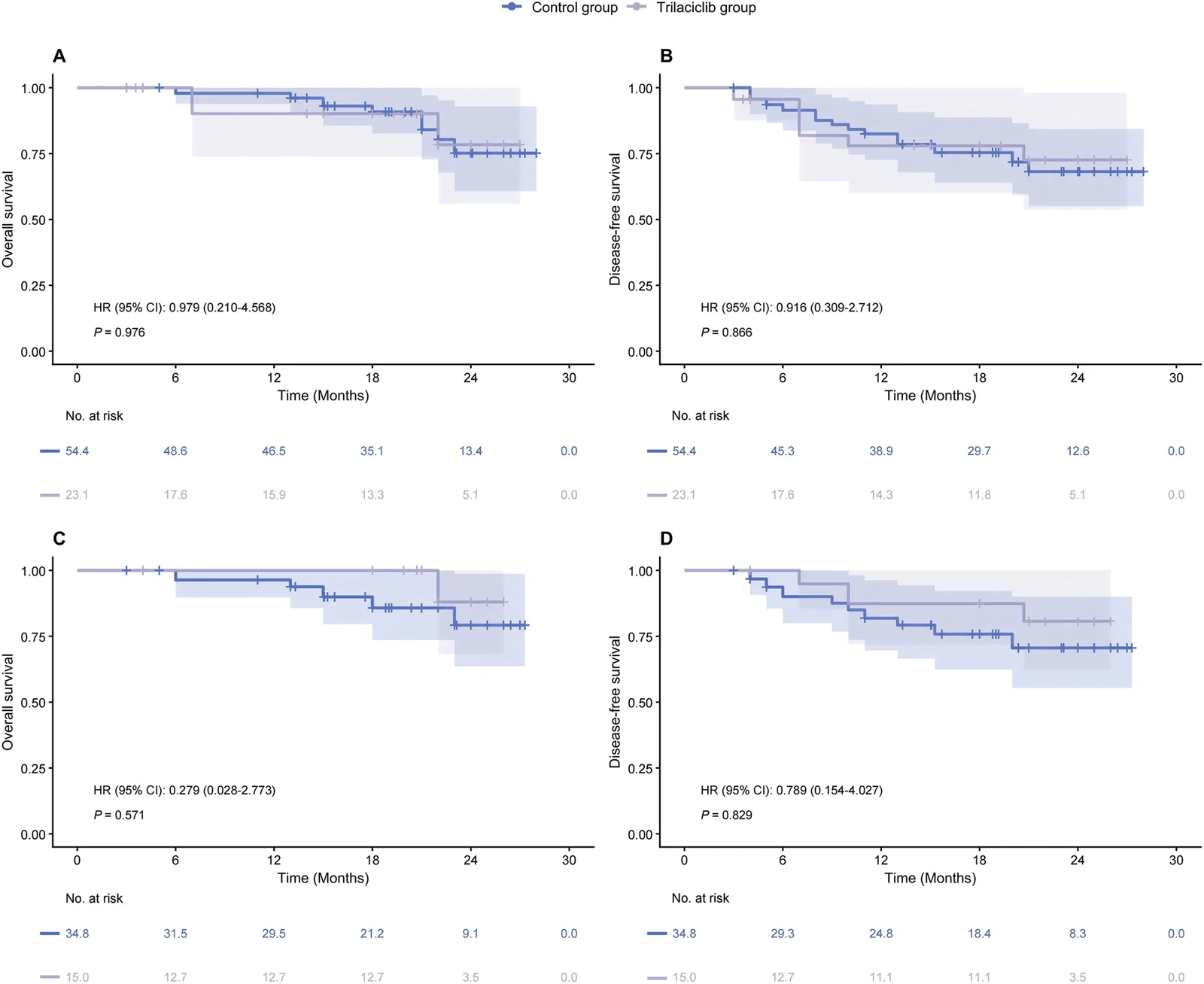
Exploratory IPTW-weighted Kaplan-Meier analyses of OS and DFS in the Control and Trilaciclib groups. **(A)** OS and **(B)** DFS in the perioperative cohort; **(C)** OS and **(D)** DFS in the nICT subgroup. All curves, HRs, and risk-table entries are IPTW-weighted; shaded areas represent 95% CIs. P values are from likelihood-ratio tests of IPTW-weighted Cox models. In the perioperative cohort, median follow-up was 21 months (IQR 15–23.3) in the control group versus 20.7 months (IQR 14.2–22) in the trilaciclib group, with 8 deaths and 14 recurrences versus 2 deaths and 5 recurrences, respectively. In the nICT subgroup, median follow-up was 19.2 months (IQR 15–23.1) versus 22 months (IQR 19–23), with five deaths and nine recurrences versus one death and three recurrences, respectively. Because of the small number of events and wide CIs, these analyses are exploratory; the absence of statistically significant differences should not be interpreted as evidence of equivalence or non-inferiority. The proportional-probability assumption was assessed using Schoenfeld residuals (all P > 0.05). Abbreviations: OS, overall survival; DFS, disease-free survival; nICT, neoadjuvant immunochemotherapy; IPTW, inverse probability of treatment weighting; HR, hazard ratio; CI, confidence interval.

In the nICT subgroup, 5 deaths and 9 DFS events occurred in the control group, compared with 1 death and 3 DFS events in the trilaciclib group. OS (HR, 0.279; 95% CI, 0.028–2.773; *P* = 0.571) and DFS (HR, 0.789; 95% CI, 0.154–4.027; *P* = 0.829) did not differ significantly between groups. Schoenfeld residual tests indicated no violation of the proportional hazards assumption for any model (all *P* > 0.05; Supplementary Figure S4). These survival analyses are exploratory, were not adjusted for multiplicity, and should not be interpreted as evidence of equivalence or noninferiority; confidence intervals were wide, reflecting the small sample size and limited follow-up duration.

## 4 Discussion

In this single-center, prospective observational cohort, we used stabilized IPTW to balance measurable baseline covariates and conducted an exploratory evaluation of myeloprotection-related outcomes associated with trilaciclib administration during perioperative immunochemotherapy in resectable ESCC. The principal findings were as follows: trilaciclib use was associated with lower incidences of grade 3–4 CIM, neutropenia, and anemia, and with lower use of hematopoietic supportive care (G-CSF and ESAs). Within the limited sample size and follow-up period, no statistically significant unfavorable differences were observed in R0 resection rate, pathologic response, or survival outcomes; however, confidence intervals were wide, and clinically meaningful benefit or harm cannot be excluded. Importantly, these results represent observational associations and cannot establish causality; their primary role is to generate hypotheses for randomized trials.

For locally advanced ESCC, neoadjuvant therapy plus surgery is an important treatment strategy, supported by the NEOCRTEC5010 and CROSS trials (Yang et al., 2018; Eyck et al., 2021). The randomized phase 3 ESCORT-NEO trial and recent comparative studies have shown that neoadjuvant immunochemotherapy improves pathologic response with acceptable safety (Qin et al., 2024; Guo et al., 2025; Wang et al., 2025). Multimodal strategies such as induction immunochemotherapy combined with radiotherapy are also being explored (Ma and Baran, 2025; Chen et al., 2026). Whether neoadjuvant immunochemotherapy or neoadjuvant chemoradiotherapy is superior requires confirmation in head-to-head randomized trials (Yu et al., 2024). Perioperative cohort studies have also reported acceptable surgical outcomes and survival after neoadjuvant immunochemotherapy (nICT) (Yang et al., 2025). Given the substantial disease burden of ESCC and the widespread use of immunochemotherapy in China, the potential beneficiary population for validated myeloprotective strategies would be considerable (Han et al., 2024; Qin et al., 2024). Concurrently, treatment intensification is accompanied by substantial hematologic toxicity, which can cause dose delays and reductions, decrease relative dose intensity (RDI), and potentially postpone surgery (Qiao et al., 2022; Crawford et al., 2024). In this evolving perioperative landscape, maintaining treatment intensity without compromising antitumor efficacy is a key challenge that supportive care must address.

Trilaciclib transiently blocks the HSPC cell cycle before chemotherapy exposure, a mechanism that supports multilineage protection (He et al., 2017; O'Leary et al., 2016; Bisi et al., 2016). Randomized studies and pooled analyses in extensive-stage small cell lung cancer (ES-SCLC) have reported reductions in CIM and hematopoietic supportive care use (Ferrarotto et al., 2021; Weiss et al., 2019). The Chinese TRACES study and recent meta-analyses—including trial sequential analysis and pooled analyses in solid tumors—have reported broadly consistent findings (Cheng et al., 2024; Ding et al., 2025; Yang et al., 2026; Ahmed et al., 2026). Small real-world studies in esophageal cancer have also suggested feasibility (Chen et al., 2025; Yan et al., 2025). The present study extends this evidence to the setting of perioperative immunochemotherapy combined with MIE. The direction of the primary outcome was consistent with the above randomized studies, though the point estimates were larger, which may reflect small-sample effect inflation, population differences, or residual confounding; direct cross-study comparison is therefore not appropriate. Yan et al., in a propensity score-matched study of concurrent chemoradiotherapy for ESCC, also reported an association between trilaciclib use and lower myelosuppression (Yan et al., 2025). Our study complements that work by covering the perioperative pathway of immunochemotherapy plus surgery. Translational studies have also suggested that CDK4/6 inhibition may enhance antitumor immunity, and related signals have been observed in metastatic triple-negative breast cancer (Tan et al., 2019; Tan et al., 2023); whether such an effect exists in the perioperative ESCC setting remains unclear. In addition, evidence from advanced ES-SCLC and breast cancer derives from relatively homogeneous populations receiving standard regimens, whereas perioperative ESCC involves more heterogeneous regimens and surgical pathways, and the curative intent makes preservation of treatment intensity more critical. Therefore, evidence from the advanced setting cannot be directly extrapolated (Zhang et al., 2025; Cheng et al., 2024).

Results varied across hematologic lineages and warrant cautious interpretation. The between-group differences in grade 3–4 neutropenia and anemia were statistically significant, whereas those in SN and FN were not. The latter two endpoints had very few events and thus provided limited power to detect between-group differences; moreover, prophylactic G-CSF was used more commonly in the control group, which may have narrowed the between-group gap in event rates. Accordingly, supportive care use serves as a supplementary endpoint reflecting the management burden of myelosuppression. The seven supplementary sensitivity analyses showed consistent directions for the primary outcome, and in the Bayesian analysis the posterior probabilities of benefit for CIM, neutropenia, and anemia all exceeded 97%. The directions of the longitudinal main effects were also unchanged after adjusting for prophylactic G-CSF. For anemia, neither the main effect of group nor the group-by-time interaction for longitudinal hemoglobin was statistically significant. The association with fewer grade 3–4 anemia events was not accompanied by statistically significant between-group differences in cycle-specific hemoglobin values in the overall cohort; these findings describe different aspects of the observed hemoglobin distribution and do not establish a selective effect on severe erythroid suppression. The statistically significant group-by-time interaction for platelets suggests that the platelet trajectories of the two groups differed over treatment cycles, consistent with the mechanism of trilaciclib protecting megakaryocytic progenitors. However, baseline platelet counts were imbalanced before weighting (SMD = 0.155); although this was corrected by weighting and targeted adjustment, this finding remains exploratory. In the longitudinal analysis, between-group differences predominantly occurred at cycles 2–4, consistent with the time window in which cumulative chemotherapy toxicity emerges. This suggests that the protective effect of trilaciclib may be more important under repeated chemotherapy exposure, consistent with the hypothesis that HSPCs are spared from cyclic injury (He et al., 2017; Bisi et al., 2016).

Notably, cycle-specific estimates from the full-cohort exposure-axis analysis (Figure 3; Table 7) were directionally and quantitatively consistent with the phase-homogeneous neoadjuvant analysis (Figure 4; Table 4), with concordant directions for neutrophils and platelets, suggesting that the main longitudinal findings were robust to cycle-phase composition. The platelet group-by-time interaction was observed only in the exposure-axis analysis (*P* = 0.044) and was not statistically significant in the phase-homogeneous analysis; it was therefore interpreted as an exploratory signal rather than confirmatory evidence.

**TABLE 7:** IPTW-weighted longitudinal analysis of hematologic parameters.

Variables	Time	Raw n (control/trilaciclib)	Weighted median (IQR) (control)	Weighted median (IQR) (trilaciclib)	Adjusted mean difference (95% CI)	*P* (FDR-adjusted)
Leukocyte	C1	65/28	5.86 (5.06, 7.10)	7.20 (6.52, 7.89)	0.86 (−0.08–1.79)	0.072 (0.096)
	C2	65/28	5.61 (4.51, 6.70)	7.12 (5.89, 8.45)	1.32 (0.64–2.00)	<0.001 (0.001)
	C3	51/22	4.20 (3.21, 5.79)	6.21 (6.10, 8.65)	1.94 (0.99–2.90)	<0.001 (0.001)
	C4	19/13	4.21 (1.94, 5.10)	6.78 (6.45, 8.70)	2.41 (0.93–3.89)	0.001 (0.004)
Neutrophil	C1	65/28	3.68 (2.97, 4.93)	4.69 (4.21, 5.48)	0.53 (−0.39–1.46)	0.260 (0.277)
	C2	64/28	3.40 (2.75, 4.64)	4.50 (3.47, 5.64)	0.87 (0.29–1.46)	0.004 (0.009)
	C3	51/22	2.87 (1.63, 3.64)	4.32 (3.54, 5.60)	1.38 (0.61–2.15)	<0.001 (0.002)
	C4	19/13	2.81 (1.42, 3.40)	4.56 (4.20, 6.12)	1.39 (0.13–2.66)	0.031 (0.055)
Hemoglobin	C1	65/28	124.00 (110.00, 134.00)	124.00 (119.00, 129.00)	1.73 (−2.80–6.26)	0.454 (0.454)
	C2	65/28	117.00 (106.00, 129.00)	124.00 (112.00, 134.00)	7.43 (−0.44–15.29)	0.064 (0.093)
	C3	51/22	112.00 (102.00, 121.00)	113.00 (108.00, 125.00)	7.44 (0.86–14.02)	0.027 (0.053)
	C4	19/13	105.00 (87.00, 113.00)	118.00 (107.00, 121.00)	10.84 (0.72–20.95)	0.036 (0.057)
Platelet	C1	65/28	238.00 (187.00, 276.00)	232.00 (215.00, 301.00)	15.92 (−5.90–37.73)	0.153 (0.180)
	C2	65/28	210.00 (178.00, 241.00)	234.00 (197.00, 275.00)	19.94 (−7.72–47.60)	0.158 (0.180)
	C3	51/22	182.00 (144.00, 213.00)	241.00 (210.00, 299.00)	65.13 (28.94–101.32)	<0.001 (0.002)
	C4	19/13	192.00 (162.00, 221.00)	221.00 (215.00, 312.00)	65.21 (20.94–109.48)	0.004 (0.009)

IPTW-weighted longitudinal analysis of hematologic parameters in the perioperative cohort (Trilaciclib vs. Control). Data are IPTW-weighted estimates from a pseudo-population and are not actual patient counts. Time points C1–C4 correspond to treatment cycles 1–4, respectively. Raw n indicates the actual number of patients with available data at each cycle. Adjusted mean differences (95% CIs) at each cycle were derived from weighted GEE models adjusting for the covariates described in the Methods. FDR-adjusted P values are shown for cycle-specific comparisons (Benjamini-Hochberg procedure applied across the four time points within each marker). Effective sample sizes were 63.3 for the control group and 25.2 for the trilaciclib group. Overall GEE effects are reported in Supplementary Table S4. BUnits: leukocyte and neutrophil counts, ×109 cells/L; hemoglobin, g/L; platelet count, ×109/L. Because of the small sample size, results should be interpreted as exploratory. Abbreviations: IPTW, inverse probability of treatment weighting; GEE, generalized estimating equations; IQR, interquartile range; FDR, false discovery rate; CI, confidence interval.

The observed lower use of supportive care is consistent with previous literature. Prophylactic G-CSF is widely used to prevent febrile neutropenia, but its effect is limited to the granulocytic lineage (Hart et al., 2023). Trilaciclib protects the more upstream HSPCs, and previous studies have shown that it reduces the use of G-CSF, ESAs, and transfusions in patients with SCLC (Ferrarotto et al., 2021; Hart et al., 2023). However, initiation of supportive care in this study remained subject to physician judgment, so this endpoint can only partly reflect the management burden of myelosuppression and should be interpreted with caution. Compared with other myeloprotective strategies, trilaciclib is characterized by administration before chemotherapy, multilineage coverage, and reversible action (Dhillon, 2021; O'Leary et al., 2016). G-CSF covers only the granulocytic lineage, and ESAs have a slow onset; neither can prevent thrombocytopenia (Hart et al., 2023). Lower use of supportive care may also reduce the burden of injections, monitoring, and hospitalization, though health economic indicators were not collected in this study, and this inference requires further validation (Crawford et al., 2024). Use of G-CSF and ESAs reflects both the occurrence of myelosuppression and the implementation of prophylactic strategies; adherence to identical initiation criteria in both groups attenuated the influence of management differences. Treatment delays attributable to myelosuppression may also postpone surgery and reduce RDI. In this study, treatment delay did not differ significantly between groups, but RDI was not systematically collected, limiting our ability to assess the full impact of trilaciclib on treatment intensity (Crawford et al., 2024).

A critical premise for applying myeloprotective agents in the perioperative setting is that antitumor efficacy remains uncompromised. No statistically significant between-group differences were observed in this study for R0 resection rate, pCR, MPR, pathologic stage, OS, or DFS, findings concordant with previous randomized studies and their follow-up (Cheng et al., 2024; Weiss et al., 2019; Tan et al., 2019; Tan et al., 2023). However, survival events were sparse (10 deaths and 19 DFS events in total), and the pathologic analysis included only 52 patients. Therefore, the absence of significant differences constitutes only a preliminary safety signal and cannot establish equivalence or demonstrate that efficacy is unaffected. The present study cannot determine whether trilaciclib modifies the antitumor effects of immune checkpoint inhibition (Tan et al., 2019). Future studies may incorporate longitudinal immunophenotyping and peripheral blood biomarker monitoring to distinguish the established myeloprotective effect from potential immunomodulatory effects (Yu et al., 2024; Guo et al., 2026). An ideal confirmatory study would use a multicenter, randomized, controlled design, with grade 3–4 CIM as the primary endpoint and RDI as a key secondary endpoint, incorporating prespecified stratification by chemotherapy regimen and immunotherapeutic agent, and including patient-reported outcomes, hospitalization, and health economic indicators; peripheral blood biomarkers could be used to explore stratification of the benefiting population (Guo et al., 2026). With a median follow-up of approximately 21 months, this remains early for a perioperative ESCC study; recurrence and death events will continue to accumulate, and long-term safety and efficacy signals require continued follow-up.

Several limitations warrant acknowledgment. First, this single-center, non-randomized study cannot fully exclude unmeasured confounding or selection bias, despite stabilized IPTW, adjustment for residual imbalance, and sensitivity analyses. Second, the sample size was small (28 patients in the trilaciclib group and 21 in the nICT subgroup), limiting power for FN, SN, pathologic, and survival outcomes; zero events do not imply zero risk, and confidence intervals were wide. Third, 19 patients had incomplete follow-up; although 15 with post-baseline data were retained, attrition bias cannot be excluded, and survival data remain immature. Fourth, the study was not registered; although not mandatory for observational studies, non-registration may introduce *post hoc* selection risk, which we mitigated with a prespecified CRF, analysis plan, and STROBE reporting. Fifth, heterogeneity in immunotherapeutic agents and perioperative pathways could not be addressed by stratified analyses; RDI, dose-reduction rates, and surgical safety endpoints were not systematically collected. Sixth, the longitudinal exposure axis pooled preoperative and postoperative cycles; because surgery may alter hematologic status, cycle-level findings require caution, with the phase-homogeneous nICT analysis providing supportive validation. Seventh, the propensity score model included baseline covariates only and did not account for time-varying factors. Finally, single-center generalizability requires multicenter validation.

## 5 Conclusion

Among patients with resectable ESCC receiving perioperative immunochemotherapy, trilaciclib use was associated with a lower incidence of grade 3–4 myelosuppression and lower use of hematopoietic supportive care. No statistically significant unfavorable differences in pathologic response or survival outcomes were observed; however, this small observational study cannot establish that antitumor efficacy was unaffected or that outcomes were equivalent. These findings are hypothesis-generating and should not inform routine clinical practice. Validation in larger, multicenter randomized controlled trials with RDI as a key secondary endpoint is needed. The effect estimates from this cohort may provide preliminary parameters for sample-size planning but should not alone determine them.

## Data Availability

The datasets presented in this article are not readily available because the data are not publicly available due to patient privacy restrictions. De-identified data are available from the corresponding author upon reasonable request. Requests to access the datasets should be directed to Hansheng Wu, wu-han-sheng@163.com.
