# Supplementary material for: Association of trilaciclib use with hematologic outcomes during perioperative immunochemotherapy in resectable esophageal squamous cell carcinoma: a prospective observational cohort study
Source: Front Pharmacol. 2026 Oct 28;17:1933364. doi: 10.3389/fphar.2026.1933364 (PMC13752184; doi:10.3389/fphar.2026.1933364)
Supplement: Supplementary file 1 [file Table1.docx]

| **Supplementary Table S1. Patient disposition, timing of incomplete follow-up, and availability of hematologic data according to treatment group.** | | | | |
| --- | --- | --- | --- | --- |
| **Patient disposition** | **Overall**  **(n = 97)** | **Immunochemotherapy alone (n = 65), n; cycle*** | **Trilaciclib plus immunochemotherapy ( n = 28), n; cycle*** | **Data included in the safety analysis** |
| Complete follow-up | 78 | 56 | 22 | All available hematologic data |
| Incomplete follow-up | 19 | 13 | 6 | See categories below |
| Transferred to another institution | 10 | 5; Cycle 1 (n = 1), Cycle 2 (n =1 ), Cycle 4 (n = 3) | 5; Cycle 2 (n = 2), Cycle 3 (n = 2), Cycle 4 (n = 1) | Hematologic and non-hematologic toxicity data collected before transfer |
| Treatment regimen modification | 5 | 4; Cycle 2 (n = 1), Cycle 3 (n = 3 ) | 1; Cycle 3 (n = 1 ) | Hematologic and non-hematologic toxicity data collected before treatment regimen modification |
| Lost to follow-up with no evaluable post-baseline data | 4 | 4 | 0 | None |
| Included in the analysis set | 93 | 65 | 28 | Available hematologic and non-hematologic toxicity data from 93 patients |
| Data are presented as numbers of patients. *Cycle number refers to the treatment cycle at the time of transfer or regimen modification. Of the 19 patients with incomplete follow-up, 15 (10 transferred, 5 with regimen modification) had evaluable post-baseline hematologic data and were included in the safety analysis; the remaining 4 patients, who were lost to follow-up with no evaluable post-baseline data, were excluded. Thus, the final safety analysis set comprised 93 patients (78 with complete follow-up and 15 with incomplete follow-up). For patients transferred to another institution, safety data collected before transfer were included. For patients whose treatment regimen was modified, safety data collected before modification were included. The categories of incomplete follow-up were mutually exclusive. | | | | |

| **Supplementary Table S2. CTCAE v5.0 grading criteria for hematologic toxicities.** | | | | |
| --- | --- | --- | --- | --- |
| **Toxicity** | **Grade 1** | **Grade 2** | **Grade 3** | **Grade 4** |
| Leukopenia | WBC < LLN–3.0 | WBC < 3.0–2.0 | WBC < 2.0–1.0 | WBC < 1.0 |
| Neutropenia | ANC < LLN–1.5 | ANC < 1.5–1.0 | ANC < 1.0–0.5 | ANC < 0.5 |
| Anemia | Hb < LLN–10.0 | Hb < 10.0–8.0 | Hb < 8.0 or transfusion indicated | Life-threatening; urgent intervention indicated |
| Thrombocytopenia | PLT < LLN–75 | PLT < 75–50 | PLT < 50–25 | PLT < 25 |
| WBC, ANC, and PLT are expressed as ×10⁹/L; Hb is expressed as g/dL. LLN, lower limit of normal; WBC, white blood cell count; ANC, absolute neutrophil count; Hb, hemoglobin; PLT, platelet count; CTCAE, Common Terminology Criteria for Adverse Events. | | | | |

| **Supplementary Table S3. Sensitivity analyses of hematologic outcomes.** | | | | | | |
| --- | --- | --- | --- | --- | --- | --- |
| **Analysis strategy** | **N (ESS)** | **Events (Control / Trilaciclib)** | **RD (95% CI)** | **Firth OR (95% CI)** | **Bayesian OR (95% CrI)** | **Posterior P(OR < 1)** |
| **Grade 3-4 CIM** | | | | | | |
| Unadjusted | 93 | 19 / 1 | -0.257 (-0.386, -0.122) | 0.130 (0.014–0.559) | 0.110 (0.012–0.485) | 100.0% |
| **Main stabilized IPTW** | **93 (88.3)** | **19 / 1** | **-0.282 (-0.415, -0.148)** | **0.106 (0.010–0.480)** | **0.086 (0.009–0.407)** | **99.9%** |
| Untruncated stabilized IPTW | 93 (87.8) | 19 / 1 | -0.282 (-0.415, -0.149) | 0.105 (0.009–0.474) | 0.087 (0.008–0.432) | 100.0% |
| IPTW, 5th-95th percentile truncation | 93 (89.2) | 19 / 1 | -0.280 (-0.411, -0.147) | 0.108 (0.010–0.488) | 0.089 (0.010–0.422) | 100.0% |
| Overlap weighting | 93 (74.3) | 19 / 1 | -0.300 (-0.441, -0.153) | 0.118 (0.007–0.739) | 0.102 (0.007–0.670) | 99.2% |
| IPTW plus covariate adjustment | 93 (88.3) | 19 / 1 | -0.282 (-0.415, -0.148) | 0.117 (0.011–0.535) | 0.043 (0.004–0.244) | 100.0% |
| Restricted to common support | 88 (83.9) | 18 / 1 | -0.279 (-0.413, -0.147) | 0.107 (0.010–0.491) | 0.090 (0.010–0.441) | 100.0% |
| IPTW + adjustment for tumor site and platelet | 93 (88.3) | 19 / 1 | -0.282 (-0.415, -0.148) | 0.105 (0.010–0.478) | 0.078 (0.008–0.390) | 100.0% |
| **Grade 3-4 CIA** | | | | | | |
| Unadjusted | 93 | 11 / 0 | -0.169 (-0.266, -0.082) | 0.083 (0.001–0.680) | 0.064 (0.003–0.532) | 99.7% |
| **Main stabilized IPTW** | **93 (88.3)** | **11 / 0** | **-0.173 (-0.273, -0.083)** | **0.082 (0.001–0.669)** | **0.064 (0.003–0.535)** | **99.6%** |
| Untruncated stabilized IPTW | 93 (87.8) | 11 / 0 | -0.173 (-0.273, -0.083) | 0.081 (0.001–0.661) | 0.066 (0.003–0.503) | 99.8% |
| IPTW, 5th-95th percentile truncation | 93 (89.2) | 11 / 0 | -0.174 (-0.274, -0.083) | 0.082 (0.001–0.670) | 0.064 (0.002–0.536) | 99.8% |
| Overlap weighting | 93 (74.3) | 11 / 0 | -0.182 (-0.288, -0.087) | 0.106 (0.001–1.166) | 0.086 (0.003–0.967) | 97.7% |
| IPTW plus covariate adjustment | 93 (88.3) | 11 / 0 | -0.173 (-0.273, -0.083) | 0.112 (0.001–0.867) | 0.047 (0.002–0.478) | 99.7% |
| Restricted to common support | 88 (83.9) | 11 / 0 | -0.187 (-0.299, -0.090) | 0.075 (0.001–0.610) | 0.061 (0.003–0.496) | 99.7% |
| IPTW + adjustment for tumor site and platelet | 93 (88.3) | 11 / 0 | -0.173 (-0.273, -0.083) | 0.083 (0.001–0.677) | 0.063 (0.003–0.515) | 99.8% |
| **Grade 3-4 CIT** | | | | | | |
| Unadjusted | 93 | 3 / 0 | -0.046 (-0.104, 0.000) | 0.313 (0.002–3.389) | 0.199 (0.006–2.323) | 89.1% |
| **Main stabilized IPTW** | **93 (88.3)** | **3 / 0** | **-0.047 (-0.108, 0.000)** | **0.311 (0.002–3.330)** | **0.194 (0.005–2.136)** | **90.0%** |
| Untruncated stabilized IPTW | 93 (87.8) | 3 / 0 | -0.047 (-0.108, 0.000) | 0.307 (0.002–3.292) | 0.198 (0.006–2.315) | 88.7% |
| IPTW, 5th-95th percentile truncation | 93 (89.2) | 3 / 0 | -0.047 (-0.108, 0.000) | 0.311 (0.002–3.337) | 0.198 (0.006–2.368) | 88.8% |
| Overlap weighting | 93 (74.3) | 3 / 0 | -0.051 (-0.118, 0.000) | 0.329 (0.002–6.858) | 0.248 (0.007–4.549) | 82.1% |
| IPTW plus covariate adjustment | 93 (88.3) | 3 / 0 | -0.047 (-0.108, 0.000) | 0.462 (0.008–3.184) | 0.175 (0.007–2.489) | 89.5% |
| Restricted to common support | 88 (83.9) | 3 / 0 | -0.051 (-0.120, 0.000) | 0.286 (0.002–3.073) | 0.185 (0.008–2.184) | 90.0% |
| IPTW + adjustment for tumor site and platelet | 93 (88.3) | 3 / 0 | -0.047 (-0.108, 0.000) | 0.336 (0.002–3.445) | 0.202 (0.007–2.401) | 88.2% |
| **Grade 3-4 CIN** | | | | | | |
| Unadjusted | 93 | 17 / 1 | -0.226 (-0.350, -0.098) | 0.151 (0.016–0.655) | 0.123 (0.015–0.562) | 99.9% |
| **Main stabilized IPTW** | **93 (88.3)** | **17 / 1** | **-0.253 (-0.387, -0.124)** | **0.122 (0.011–0.553)** | **0.104 (0.011–0.486)** | **99.9%** |
| Untruncated stabilized IPTW | 93 (87.8) | 17 / 1 | -0.253 (-0.387, -0.124) | 0.120 (0.011–0.547) | 0.099 (0.010–0.466) | 99.9% |
| IPTW, 5th-95th percentile truncation | 93 (89.2) | 17 / 1 | -0.251 (-0.382, -0.124) | 0.123 (0.011–0.562) | 0.100 (0.010–0.478) | 99.9% |
| Overlap weighting | 93 (74.3) | 17 / 1 | -0.272 (-0.416, -0.130) | 0.133 (0.008–0.845) | 0.115 (0.009–0.758) | 98.8% |
| IPTW plus covariate adjustment | 93 (88.3) | 17 / 1 | -0.253 (-0.387, -0.124) | 0.136 (0.013–0.633) | 0.050 (0.005–0.314) | 100.0% |
| Restricted to common support | 88 (83.9) | 16 / 1 | -0.248 (-0.376, -0.121) | 0.124 (0.011–0.573) | 0.101 (0.010–0.499) | 99.9% |
| IPTW + adjustment for tumor site and platelet | 93 (88.3) | 17 / 1 | -0.253 (-0.387, -0.124) | 0.118 (0.011–0.545) | 0.088 (0.009–0.418) | 100.0% |
| **Febrile neutropenia** | | | | | | |
| Unadjusted | 93 | 6 / 1 | -0.057 (-0.153, 0.047) | 0.499 (0.050–2.544) | 0.391 (0.036–2.102) | 84.4% |
| **Main stabilized IPTW** | **93 (88.3)** | **6 / 1** | **-0.072 (-0.176, 0.028)** | **0.402 (0.034–2.104)** | **0.311 (0.026–1.697)** | **90.1%** |
| Untruncated stabilized IPTW | 93 (87.8) | 6 / 1 | -0.073 (-0.176, 0.028) | 0.397 (0.034–2.079) | 0.301 (0.024–1.737) | 90.5% |
| IPTW, 5th-95th percentile truncation | 93 (89.2) | 6 / 1 | -0.069 (-0.168, 0.029) | 0.415 (0.035–2.188) | 0.313 (0.027–1.849) | 88.9% |
| Overlap weighting | 93 (74.3) | 6 / 1 | -0.075 (-0.189, 0.033) | 0.414 (0.024–3.753) | 0.337 (0.020–3.067) | 82.0% |
| IPTW plus covariate adjustment | 93 (88.3) | 6 / 1 | -0.072 (-0.176, 0.028) | 0.533 (0.043–2.644) | 0.252 (0.018–2.001) | 90.0% |
| Restricted to common support | 88 (83.9) | 5 / 1 | -0.052 (-0.141, 0.045) | 0.498 (0.042–2.817) | 0.379 (0.034–2.439) | 83.6% |
| IPTW + adjustment for tumor site and platelet | 93 (88.3) | 6 / 1 | -0.072 (-0.176, 0.028) | 0.352 (0.024–2.086) | 0.255 (0.022–1.689) | 92.1% |
| **Severe neutropenia** | | | | | | |
| Unadjusted | 93 | 8 / 1 | -0.087 (-0.189, 0.024) | 0.369 (0.038–1.766) | 0.282 (0.034–1.532) | 92.2% |
| **Main stabilized IPTW** | **93 (88.3)** | **8 / 1** | **-0.111 (-0.221, -0.001)** | **0.284 (0.025–1.402)** | **0.215 (0.017–1.237)** | **95.5%** |
| Untruncated stabilized IPTW | 93 (87.8) | 8 / 1 | -0.111 (-0.221, -0.002) | 0.281 (0.024–1.385) | 0.222 (0.023–1.107) | 96.5% |
| IPTW, 5th-95th percentile truncation | 93 (89.2) | 8 / 1 | -0.108 (-0.217, -0.000) | 0.291 (0.025–1.443) | 0.219 (0.022–1.144) | 96.2% |
| Overlap weighting | 93 (74.3) | 8 / 1 | -0.123 (-0.246, 0.001) | 0.291 (0.017–2.247) | 0.233 (0.014–2.005) | 90.9% |
| IPTW plus covariate adjustment | 93 (88.3) | 8 / 1 | -0.111 (-0.221, -0.001) | 0.383 (0.038–1.776) | 0.158 (0.014–1.054) | 97.2% |
| Restricted to common support | 88 (83.9) | 7 / 1 | -0.094 (-0.199, 0.014) | 0.325 (0.028–1.668) | 0.256 (0.025–1.365) | 94.2% |
| IPTW + adjustment for tumor site and platelet | 93 (88.3) | 8 / 1 | -0.111 (-0.221, -0.001) | 0.256 (0.020–1.358) | 0.189 (0.018–1.081) | 96.9% |
| Stabilized IPTW was used to balance baseline covariates. Eight prespecified analysis strategies were applied, including unadjusted, various weight truncations, overlap weighting, and IPTW plus covariate adjustment. RD (with bootstrap 95% CI) was calculated as the weighted event proportion difference. Firth-penalized logistic regression and Bayesian logistic models (with weakly informative priors) were used to address sparse-data and zero-event outcomes. ESS = (Σw)² / Σw². N denotes the unweighted evaluable patient count. Posterior probability of benefit [P(OR < 1)] indicates the probability that trilaciclib reduces the risk of the outcome. Abbreviations: CI, confidence interval; CIA/CIM/CIN/CIT, chemotherapy-induced anemia/myelosuppression/neutropenia/thrombocytopenia; CrI, credible interval; ESS, effective sample size; FN, febrile neutropenia; IPTW, inverse probability of treatment weighting; OR, odds ratio; RD, risk difference; SN, severe neutropenia. | | | | | | |

| **Supplementary Table S4. Overall GEE effects for treatment, time, and treatment-by-time interaction in the perioperative cohort and the nICT subgroup.** | | | | | |
| --- | --- | --- | --- | --- | --- |
| **Perioperative cohort** | | |  | **nICT subgroup** | |
| **Variables** | **Overall effect** | ***P*** value (FDR-adjusted) |  | **Overall effect** | ***P*** value (FDR-adjusted) |
| Leukocyte | Treatment | 0.108 |  | Treatment | 0.015 |
|  | Time | <0.001 |  | Time | <0.001 |
|  | Treatment x Time | 0.227 |  | Treatment x Time | 0.506 |
| Neutrophil | Treatment | 0.389 |  | Treatment | 0.057 |
|  | Time | <0.001 |  | Time | <0.001 |
|  | Treatment x Time | 0.397 |  | Treatment x Time | 0.774 |
| Hemoglobin | Treatment | 0.454 |  | Treatment | 0.602 |
|  | Time | <0.001 |  | Time | <0.001 |
|  | Treatment x Time | 0.405 |  | Treatment x Time | 0.122 |
| Platelet | Treatment | 0.153 |  | Treatment | 0.722 |
|  | Time | <0.001 |  | Time | <0.001 |
|  | Treatment x Time | 0.044 |  | Treatment x Time | 0.280 |
| P values are from Wald tests based on the weighted GEE models adjusted for treatment, time, treatment-by-time interaction, baseline value, and tumor location. To account for multiple comparisons, P values for the three effects (Treatment, Time, and Treatment × Time) within each marker were adjusted using the Benjamini-Hochberg procedure to control the false discovery rate. Abbreviations: GEE, generalized estimating equations; nICT, neoadjuvant immunochemotherapy; FDR, false discovery rate. | | | | | |

| **Supplementary Table S5. Sensitivity analysis: GEE models with additional adjustment for prophylactic G-CSF use.** | | | | |
| --- | --- | --- | --- | --- |
| **Variables** | **Primary model: Treatment *P*** | **Adjusted for G-CSF: Treatment *P*** | **Primary model: Treatment x Time *P*** | **Adjusted for G-CSF: Treatment x Time *P*** |
| Leukocyte | 0.072 | 0.018 | 0.226 | 0.230 |
| Neutrophil | 0.260 | 0.046 | 0.397 | 0.316 |
| Hemoglobin | 0.451 | 0.278 | 0.222 | 0.219 |
| Platelet | 0.153 | 0.092 | 0.029 | 0.029 |
| Data were analyzed using weighted GEE models with an exchangeable working correlation structure, IPTW, and adjustment for baseline outcome value, tumor location, and prophylactic G-CSF use. G-CSF use was defined as ever versus never during the study treatment period. P values were obtained from Wald tests. G-CSF use was not included in the primary model because it may occur after treatment initiation and may lie on the pathway between trilaciclib and hematologic outcomes. This analysis should therefore be interpreted as a sensitivity analysis assessing the robustness of the treatment association after additional adjustment for G-CSF use, rather than as a formal mediation or direct-effect analysis. Given the observational design and limited sample size, all findings are exploratory. | | | | |
